# Environmentally derived subgroups of preadolescents with family history of substance use exhibit distinct patterns of psychopathology and reward-related behaviors: insights from the ABCD study

**DOI:** 10.3389/frcha.2025.1631474

**Published:** 2025-11-13

**Authors:** Srinivasan A. Ramakrishnan, Riaz B. Shaik, Siddhartha Peri, Faith Adams, Shalaila S. Haas, Sophia Frangou, Shankar Srinivasan, Omar El-Shahawy, Christopher J. Hammond, Iliyan Ivanov, Muhammad A. Parvaz

**Affiliations:** 1 Department of Health Informatics, Rutgers—School of Health Professions, Piscataway, NJ, United States; 2 Department of Psychiatry, Icahn School of Medicine at Mount Sinai, New York, NY, United States; 3 Djavad Mowafaghian Centre of Brain Health, Department of Psychiatry, University of British Columbia, Vancouver, BC, Canada; 4 Department of Population Health, NYU Langone Health, New York, NY, United States; 5 Department of Psychiatry & Behavioral Sciences, Division of Child & Adolescent Psychiatry, Johns Hopkins University School of Medicine, Baltimore, MD, United States; 6 Department of Neuroscience, Icahn School of Medicine at Mount Sinai, New York, NY, United States; 7 Department of Artificial Intelligence and Human Health, Icahn School of Medicine at Mount Sinai, New York, NY, United States

**Keywords:** family history of addiction, environmental factors, impulsivity, k-means (KM) clustering, ABCD study

## Abstract

**Background:**

Family history of substance use (FHSU), along with sociodemographic and psychosocial factors, has been identified as a key risk factor for adolescent substance use and progression to substance use disorders (SUD). However, the interaction between distinct sociodemographic and psychosocial profiles in adolescents with FHSU and constitutional factors, such as psychopathological symptom severity, impulsivity, and reward processing, remains unclear. Given the complexity of these factors, it is crucial to explore how these elements contribute to the differential vulnerability to SUD among youth with family history of substance use. Particularly as, the identification of clinically relevant subgroups of at-risk youth may inform precision prevention and treatment approaches to reduce adverse outcomes related to SUDs.

**Methods:**

Here, we used data from the Adolescent Brain Cognitive Development (ABCD) study and grouped the participants (age: 9–10 years) into positive and negative FHSU [i.e., FHSU-P (*n* = 1955; female 49.7%, White 57.95%), and FHSU-N (*n* = 4,369; female 48.33%, White 61.16%), respectively]. We used K-means clustering to identify latent subgroups in the FHSU-P population based on psychosocial variables and then compared the resulting subgroups on internalizing, externalizing, and total psychopathology, impulsivity, and reward prediction errors.

**Results:**

K-means clustering revealed five subgroups within FHSU-P: Subgroups 1 (*n* = 744) and 2 (*n* = 300) exhibited favorable psychosocial profiles, marked by higher school involvement, social engagement, and parental acceptance. Subgroups 3 (*n* = 267), 4 (*n* = 201), and 5 (*n* = 443) were characterized by lower engagement across peer, school, and parental domains. Group comparisons showed that Subgroups 1 and 2 had comparable levels of psychopathology and impulsivity, while Subgroups 3, 4, and 5 displayed higher psychopathology and impulsivity. Reward prediction errors were similar across all subgroups. Other group differences are also presented and discussed in the main text.

**Conclusion:**

These findings highlight significant heterogeneity within the FHSU-P group and emphasize the importance of stratifying adolescents based on sociodemographic and psychosocial factors. Such stratification can help identify adolescents at higher risk for psychopathologies, including SUDs, offering insights for targeted prevention and intervention strategies.

## Introduction

Pathways and mechanisms for the development of substance use disorders (SUD) in adolescents are complex and even after years of investigation remain poorly understood. Research efforts have generally been advancing in two main directions: (a) investigating purported biological and familial underpinnings of SUDs, and (b) examining environmental factors that influence the onset of SUDs. However, the interface of both constitutional [e.g., family history of substance use (FHSU)] and environmental (e.g., parenting, peer relations, neighborhood safety) aspects of adolescent substance experimentation and use remains unclear. For instance, prior research shows that FHSU positive youth (FHSU-P) are at increased risk for developing problem drug use (1, 2), and it is speculated that this elevated risk might be related to alterations in reward processing and behavioral inhibition (3, 4), or other processes generally associated with the functions of the prefrontal cortex (5). More generally, increased risk-taking, including substance use, occurs during adolescence, regardless of FHSU status, and might be linked to the imbalance in the developmental course of a rapidly developing motivation-reward system, which contributes to the pursuit of rewarding, exciting, and novel experiences and a more gradually developing cognitive control system, exercising restraint on potentially harmful impulses (6–8). Functional imbalance in the development of these systems may present clinically as high level of impulsivity, which can be measured with the appropriate psychometric assessments (9).

While FHSU is known to be a risk factor for SUD development, it is important to note that not all FHSU-P youth go on to use substances problematically or develop SUDs. Among FHSU-P youth, there are many mediating factors that may either heighten or diminish risk for SUD (10). Such protective effects can be attributed to both biological features like increased recruitment of the dorsolateral prefrontal cortex during inhibitory task (11) or increased resting state connectivity between brain regions implicated in cognitive control (12) but may also be linked to environmental influences (10). A review by Petraitis et al. (13) examined theories of adolescent substance use that focused on substance-specific conditions, social learning processes and attachment to family, and classified environmental influences into social, attitudinal and interpersonal factors. Subsequent literature has pointed out the complex nature of the interactions between personal and environmental factors in relation to both vulnerability and resilience. One review discussed the definition of resilience as “positive adaption inspite of adversity” and identified “protective” factors from three levels (e.g., family, school and community) (14) These included parental supervision, family bonding, positive peer connections and school and community engagement among others. Others have independently found that factors like “prosocial peers”, “home and school support”, and “meaningful community participation” appear to confer protective value in adolescents in relation to tobacco, alcohol and illicit drug use, however, they also noted that the beneficial effects are not universal but might be limited to a small set of factors specific to different populations (15). Indeed, exploring the family history of SUD requires examining the multidimensional and interacting pathways of personal and environmental influences, which may simultaneously contribute to risk and promote resilience, highlighting the importance of an integrative framework over isolated analyses.

Indeed, several models have been proposed to capture the interplay among various factors that contribute to SUD risk. The social development model theory presents a framework which stipulates that children learn patterns of behavior from the socializing agents of family, school, peers and various community institutions, which can affect future behaviors (16, 17), and can be used to reliably predict substance use in late adolescence (18). The Bronfenbrenner's Bioecological Model (19) examines the interactions between the person's unique characteristics, their immediate and broader societal environment and how these elements change over time. These multi-system interactions affect the development of adolescent health behavior, including substance use. In turn, the differential susceptibility hypothesis suggests that some youth are characterized by increased sensitivity to environmental contexts possibly linked to specific genetic makeup (20). Moreover, neurobiological changes that occur during adolescence as part of normative maturation of the central nervous system, may also contribute to the etiology of adolescent substance use (21–23). These models and theories suggest that a large group of young individuals who are at risk of developing SUDs (e.g., FHSU-P youth) will most likely be comprised of subgroups of individuals who share sets of environmental exposures that may influence different trajectories of engagement with drug use. This further suggests that FHSU alone may have a limited predictive value for SUD development and that more detailed stratification based on environmental and psychosocial risk factors is warranted to identify different levels of SUD risk (24–26). Taken together, examining subgroups based on environmental and psychosocial risk exposures within FHSU-P may identify individual- as well as group-level targets of intervention to decelerate the rate of substance use experimentation and use.

The Adolescent Brain and Cognition Development (ABCD) Study offers the largest cohort of young children with information about both constitutional predisposition for SUD (e.g., FHSU) as well as many indexes of environmental exposures. For example, a recent report from the ABCD cohort identified clusters of environmental exposure that showed consistent and replicable associations with multiple measures of brain organization (27). Similarly, another group found evidence that greater environmental risk and stress were associated with higher endorsement of distressing psychosis like experiences particularly among minority children from the ABCD study (28). A similar study, albeit in a small cohort, used clustering technique to identify groups based on parental substance use and examined its influence on stress and substance use vulnerability in children (29). Thus, availability of these data allows for the examination of a considerable number of interactions between FHSU and environmental exposures, and their association with key cognitive and clinical outcomes such as impulsivity, reward processing and psychopathological symptoms.

To test these interactions, in this proof-of-concept study, we used unsupervised machine learning to categorize FHSU positive (FHUS-P) youth into subgroups based on diverse sociodemographic and environmental exposures, which have previously been identified as key risk factors (30–32). Importantly, we operationalized FHSU to specifically capture only those relatives whose substance use was associated with significant functional or psychosocial impairment. Unlike broader definitions based solely on substance exposure, or formal diagnostic categories such as the family history of SUD that rely on clinical confirmation, our definition balances specificity and feasibility within a large-scale and non-clinical ABCD sample. Based on available evidence we hypothesized that a cohort of FHSU-P youth ages 9–10 from the ABCD study can be stratified into subgroups based on known sociodemographic and environmental factors using a machine learning clustering method. To compare the relative extent of risk between these subgroups, we planned to conduct group comparisons between FHSU Negative (FHSU-N) and each of the FHSU-P subgroups, as well as between FHSU-P subgroups on clinical factors such as parental behavioral reports, trait impulsivity, and reward-related behaviors. We hypothesized that the scores of these behavioral indexes will be significantly different among the subgroups and will help in distinguishing subgroups with greater and lesser risk for initiating problem substance use.

## Methods

### Participants

The study sample comprised of participants from the ABCD Study baseline cohort, with data supplemented by Year 1 follow-up assessments (Release 5.0). For this analysis, we selected 6,324 participants with complete data on all relevant variables, including sociodemographic characteristics (Table 1), impulsivity measures (Supplementary Table S1, Supplementary Online), and Clinical Behavior Checklist (CBCL) scores (Supplementary Table S2, Supplementary Online) (33). The cohort selection procedure is detailed in Supplementary Figure S1 (Supplementary Online).

**Table 1:** Demographic details and test statistics for the FHSU cohort.

Demographic details	FHSU-N	FHSU-P	Test statistics
	*N* = 4,369	*N* = 1,955	
Sex			
Male	2,250 (51.5%)	982 (50.2%)	Z = −0.893, *p* = 0.372
Female	2,110 (48.3%)	971 (49.7%)	
Other	9 (0.19%)	2 (0.1%)	
Parent age			
20–25 Years	15 (0.34%)	3 (0.15%)	Z = −5.082, *p* = < 0.001
26–30 years	229 (5.24%)	132 (6.75%)	
31–35 years	581 (13.3%)	407 (20.82%)	
36–40 Years	1,313 (30.05%)	537 (27.47%)	
41–45 Years	1,335 (30.56%)	480 (24.55%)	
46–50 Years	657 (15.04%)	291 (14.88%)	
>50 Years	217 (4.97%)	101 (5.17%)	
Race & ethnicity			
**Hispanic**	774 (17.72%)	400 (20.46%)	Z = −2.440, *p* = 0.015
White	2,672 (61.16%)	1,133 (57.95%)	
Black	422 (9.66%)	193 (9.87%)	
Asian	97 (2.22%)	8 (0.41%)	
others	404 (9.25%)	221 (11.3%)	
Parent relationship			
Child's Biological Mother	3,810 (87.21%)	1,702 (87.06%)	Z = −0.675, *p* > 0.500
Child's Biological Father	485 (11.1%)	143 (7.31%)	
Adoptive Parent	20 (0.46%)	58 (2.97%)	
Child's Custodial Parent	18 (0.41%)	29 (1.48%)	
Other	36 (0.82%)	23 (1.18%)	
Parent employment			
Working now: Full-Time/Part-Time	3,164 (72.42%)	1,436 (73.45%)	Z = −0.949, *p* = 0.343
Temporarily Laid off	24 (0.55%)	6 (0.31%)	
Looking for work	120 (2.75%)	58 (2.97%)	
Retired	14 (0.32%)	14 (0.72%)	
Disabled: Permanently or Temporarily	50 (1.14%)	50 (2.56%)	
Stay-at-Home Parent	807 (18.47%)	283 (14.48%)	
Student	71 (1.63%)	59 (3.02%)	
Other (Specify)	76 (1.74%)	34 (1.74%)	
Sick Leave	2 (0.05%)	4 (0.2%)	
Maternity Leave	4 (0.09%)	4 (0.2%)	
Unemployed not looking for work	29 (0.66%)	5 (0.26%)	
Refused to answer	8 (0.18%)	10 (0.1%)	
Marital status			
Married	3,382 (77.41%)	1,310 (67.01%)	Z = −8.716, *p* < 0.0001
Widowed	21 (0.59%)	19 (0.88%)	
Divorced	350 (8%)	206 (10.54%)	
Separated	117 (2.68%)	85 (4.35%)	
Never Married	330 (7.55%)	223 (11.41%)	
Living with Partner	155 (3.55%)	108 (5.52%)	
Refused to Answer	14 (0.32%)	4 (0.2%)	
Combined income			
Less than $5,000	95 (2.17%)	32 (1.64%)	Z = −8.892, *p* < 0.0001
$5,000 through $11,999	80 (1.83%)	48 (2.46%)	
$12,000 through $15,999	70 (1.6%)	27 (1.38%)	
$16,000 through $24,999	123 (2.82%)	76 (3.89%)	
$25,000 through $34,999	180 (4.12%)	112 (5.73%)	
$35,000 through $49,999	278 (6.36%)	199 (10.18%)	
$50,000 through $74,999	502 (11.49%)	287 (14.68%)	
$75,000 through $99,999	621 (14.21%)	332 (16.98%)	
$100,000 through $199,999	1,513 (34.63%)	575 (29.41%)	
$200,000 and greater	625 (14.31%)	168 (8.59%)	
Don't know	132 (3.02%)	53 (2.71%)	

We characterized FHSU using the parent-reported “Family History Assessment Part 1” questionnaire on Family History of Psychopathology and Substance Use (34), which specifically inquired, “*Has ANY blood relative of your child ever had any problems due to drugs? such as Marital separation or divorce; Laid off or fired from work; Arrests or DUIs; Drugs harmed their health; In a drug treatment program; Suspended or expelled from school 2 or more times; Isolated self from family, caused arguments or were high a lot*”. Children of parents who answered “Yes” were categorized as FHSU-P, while those whose parents answered “No” as FHSU-N (Supplementary Figure S1). This operationalization reflects a more stringent criterion than simple exposure, as it captures only those relatives whose substance use was associated with significant functional or psychosocial impairment.

### Measures

#### Psychopathology

We assessed participant psychopathology using t-scores from the parent-reported Child Behavior Checklist (CBCL). The CBCL is a 113-item questionnaire designed to evaluate psychiatric symptoms and behavioral problems in adolescents aged 11–18 years. Each CBCL item is rated on a three-point scale, including 0 (not true), 1 (somewhat or sometimes true), and 2 (very true or often true). The CBCL item scores were used to compute eight dimensions of internalizing and externalizing behaviors as well as the total CBCL score (35). For the current study, we used validated broad-band scales assessing symptoms of internalizing, externalizing behaviors, and total problems as outcome variables.

#### Impulsivity

Trait impulsivity was assessed using the youth-reported Short Form UPPS-P Impulsive Behavior Scale (UPPS-P), a validated 20-item version of the original 59-item questionnaire (36).This measure assesses impulsivity across five dimensions: negative urgency, positive urgency, lack of premeditation, lack of perseverance, and sensation seeking. Items were rated on a Likert scale from 1 (“agree strongly”) to 4 (“disagree strongly”), and the additive total score for each dimension was in this study. Higher scores indicate greater levels of trait impulsivity.

#### Reward prediction error

Reward prediction error was computed from the behavioral data from the Monetary Incentive Delay (MID) task (37, 38). The MID task is designed to measure and assess anticipation and outcome of gain or loss and thereby is suitable to determine positive and negative prediction error (PPE and NPE, respectively). This task has been used extensively to study the development of reward circuits (39–41) and the impact of substance use on these circuits (38, 42–47). However, some have also raised concerns that the MID is suboptimal for computing of RPE (48–50).

Each trial of the MID task begins with an incentive cue (2,000 ms) for five possible trial types (Win $.20, Win $5, Lose $.20, Lose $5, $0-no money at stake) and is followed by a jittered anticipation event (1,500–4,000 ms). Next, a variable target (150–500 ms) appears during which the participant responds to either win money or avoid losing money. This is followed by a feedback message informing the participant of the outcome of the trial. The win and lose trials are categorized as Small and Large Reward and Small and Large loss respectively. No money stake trial is categorized as neutral trial. The duration of the feedback is calculated as 2,000 ms minus the target duration. The task consists of twelve trial orders of the task (2 runs each). Each run consists of 50 contiguous trials (10 per trial type) presented in pseudorandom order and lasts 5:42 min (51).

#### Computational model

To determine the Reward Prediction Error (RPE), we used the Reinforcement Learning (RL) framework of (52, 53). This model determines the RPE using reward cues and actual reward outcomes. The model had two independent variables expected value (EV) and Reward Prediction Error (RPE). For any given trial “t”

$$EV_{t}\;\;=\;pGain_{t}\times \;Cu_{t}$$

$$RPE_{t}=\;\;R_{t}\;\;-\;EV_{t}\;$$

$$pGain_{t+1}=\;\;pGain_{t}\;+\;\eta \;\frac{RPE_{t}}{\;Cu_{t}}$$

where, Cu is the possible reward (−0.2, −5, 0, 5 or 0. 2 points), EV is the expected value (EV), R is the actual reward, RPE is the reward prediction error, pGain is participant's subjective probability of obtaining the reward, *η* is the learning rate, and t corresponds to trial t. Probability (pGain) was initially set to 0.5 and was predicated based on prediction error and cue in the subsequent trials. The learning rate was assumed to be the same for all subjects and set to 0.7 (52, 54). We determined the positive and negative prediction error (PPE and NPE, respectively). PPE is when the reward is higher than the expected value and NPE when reward is lower than the expected value.

### Statistical analysis

#### K-means clustering

K-means clustering (85) was used to identify distinct subgroups among FHSU-P adolescents. K-means clustering aims to partition, or minimize, the average squared distance between “n” observations and a centroid of a cluster, such that each observation is assigned to the cluster with the nearest mean (55). K-means clustering has been favored over other unsupervised learning methods because of its cluster quality, relative strength in predictive power in large data sets (56, 57).

In this study, K-Means clustering was performed among the FHSU- P group based on 33 socio-demographic variables (Supplementary Table S3), selected based on their established relevance to influence brain-behavior-environment associations (27). These variables spanned three broad domains: (i) 8 parental characteristics (e.g., age, education, employment, marital status), (ii) 11 family and sustenance-related indicators (e.g., income, financial adversity, psychiatric history), and (iii) 14 child and school-related factors (e.g., academic engagement, peer relationships, substance use). Missing values were imputed where required to avoid skewness in clustering analyses. This multidimensional approach was intended to capture the complex socio-environmental context surrounding each participant, enhancing the interpretability and ecological validity of the resulting clusters. The number of clusters to identify the subgroups was chosen based on the Elbow Method (58–60) that involves the computation of distortion between cluster points and their centroids. The objective is to identify the optimal number of subgroups that minimize distortion, to ensure the stability and reliability of the clustering, we ran the clustering analysis k-means (n_init = 10) across 20 random seeds, yielding identical 5-cluster solutions with minimal centroid variation (mean shift: 0.004 ± 0.002). Further Bootstrap resampling (*n* = 100) produced highly consistent cluster assignments (mean NMI: 0.941 ± 0.021, range: 0.879–0.979). Such rigorous technique yielded optimal cluster number (k = 5) which was chosen based on the elbow method (k = 2–10). To stabilize the number of subgroups, a Silhouette visualization was performed. Silhouette scores (61–63) are calculated for different cluster/subgroup configurations, and the results are analyzed to finalize the number of subgroups. Davies scores (64, 65) were used to determine the degree of separation of Subgroups and the predictive power of underlying data. Principal Component Analysis (PCA) was employed for visualizing the Subgroups and gaining insights into their structure. Finally, SHapley Additive exPlanations (SHAP) method (66) was used to assess the relative contribution of each feature to model predictions.

Additionally, we performed a sensitivity analysis, calculating family history density of SUD (FHD) scores for each participant in every subgroup and then comparing FHSU based subgroups on FHD scores, to ensure that our FHSU-P clusters were not just a proxy for density of alcohol and drug problems in the family system. For each participant, FHD was calculated by taking the sum of positive reports of problems from biological parents (+0.5) and biological grandparents (+0.25) (67). These were combined across alcohol and drug problems ranging from 0 to 4, with a score of 0 indicating the absence of problems. All participants were assigned an FHD score.

#### Mixed linear models

All CBCL, UPPS-P, and RPE measures were analyzed using linear mixed-effects models (LMM) implemented with the lme4 package (https://cran.r-project.org/web/packages/lme4/lme4.pdf) in R (http://www.r-project.org/). Subgroup membership (FHSU-P), identified via k-means clustering, was entered as a categorical fixed-effect predictor, with one subgroup designated as the reference category. Key demographic variables (sex assigned at birth, interview age, and race/ethnicity) were included as fixed-effect covariates to account for variations in biological differences, parenting style, and socio-economic context. Dependent variables included psychopathology indexed by CBCL (internalizing problems, externalizing problems, and total problem scores), impulsivity indexed by the UPPS-P subscales (positive urgency, lack of premeditation, lack of perseverance, sensation seeking, and negative urgency), and reward prediction error parameters (positive and negative) from the MID task. Random intercepts were specified for site to account for study site variability and for family ID to account for the non-independence of sibling data (*n* = 1,588 siblings, 25.11% of sample).

The general form of the model used was:

lmer(outcome_variable∼subgroup+sex+age+race_ethnicity+(1 | site)+(1 | family_ID), data=dataset, REML=FALSE)

Here, “outcome_variable” represents one of the behavioral or psychological measures; subgroup denotes the assigned FHSU-P subgroup; and sex, age, and race/ethnicity are fixed-effect covariates. Site and family ID are included as random effects. Models were fit using maximum likelihood estimation (REML = FALSE) to allow comparison of nested models.

To address multiple comparisons, false discovery rate (FDR) correction was applied within each outcome domain (CBCL, UPPS-P, and RPE) using the Benjamini–Hochberg procedure. This approach controls the false discovery rate within each family of related tests, ensuring domain-specific and statistically rigorous interpretation of results.

## Results

### Sample characteristics

Demographics of the FHSU sample, stratified by group (FHSU-N: *n* = 4,369; FHSU-P: *n* = 1,955), are summarized in Table 1. Significant differences were observed in select sociodemographic factors. The FHSU-N and FHSU-P groups did not significantly differ in sex distribution (*p* = 0.372) or parent employment status (*p* = 0.343). However, parents of FHSU-P youth were significantly younger than those of FHSU-N youth (*p* < 0.001), though most parents in both groups were between 31 and 45 years old (FHSU-N: 73.91%, FHSU-P: 72.84%).There were also significant group differences in ethnicity, with a greater proportion of Hispanic participants in the FHSU-P compared to FHSU-N group (20.46% vs. 17.72%, *p* = 0.015).

Significant group differences were also observed in marital status (*p* < 0.0001), such that there was a higher proportion of married parents in FHSU-N group (77.41%) compared to FHSU-P group (67.01%). Lastly, combined income levels also varied significantly between the two groups, with the FHSU-N group reporting a higher proportion of individuals earning $200,000 or more annually compared to those with FHSU-P (14.31% vs. 8.59%, *p* < 0.0001).

### K-means clustering

**Figure 1:**
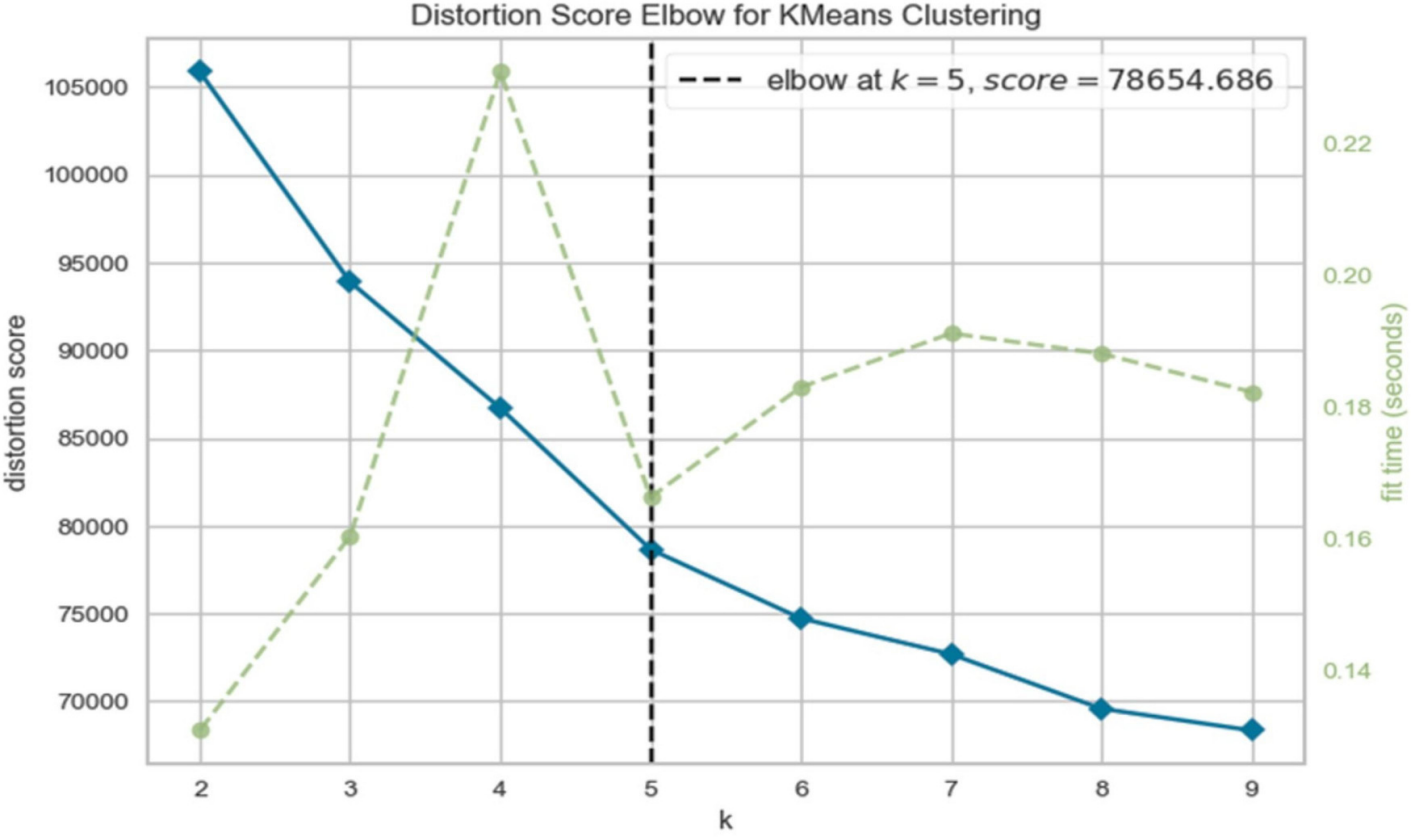
Elbow method determining the number of clusters.

Clustering of FHSU-P group using the Elbow method (Figure 1) revealed a distortion score of 78654.686 at k = 5 after which the distortion score flattened, suggesting that five clusters (subgroups) were the optimal solution. This solution was further supported by the Silhouette score (Supplementary Table S4, Supplementary Online), Davies Bouldin score (65) and the Calinski Harabaz Index.

The SHAP values defined the relative contribution of the input features in each subgroup. Across subgroups, the greatest SHAP values were noted for Involvement with prosocial peers, parent employment, parents combined income, school disengagement, school involvement, marital status, social role engagement, involvement with rule breaking peers, likeness to parent behavior. These nine factors accounted for the largest amount of variance that defined the subgroups. The subgroups, as shown in Table 2, are defined as:

- *Subgroup 1* was primarily characterized by working parents. Other factors included high positive attitude towards school, high family income and moderate acceptance of parenting.
- *Subgroup 2* was primarily characterized by the parent-responder being a stay-at-home parent. Other factors included high positive attitude towards school, high income, moderate social engagement, and moderate acceptance of parenting.
- *Subgroup 3* was primarily characterized by the lower income and not married parents. Other factors included neither positive or negative attitude towards school, low social engagement, and low acceptance of parenting styles.
- *Subgroup 4* was primarily characterized by the lowest pro-social peer involvement. Other factors included high family income, moderate positive attitude towards school, limited social engagement, and low acceptance of parenting.
- *Subgroup 5* was primarily characterized by the least likeness to school. Other factors included high income, low school involvement, low social engagement, and low acceptance of parent behavior.

**Table 2:** FHSU-P subgroups and their defining characteristics, with the highest-ranked differentiating features highlighted.

Subgroup determining factors	Subgroup 1 (*n* = 744)	Subgroup 2 (*n* = 300)	Subgroup 3 (*n* = 267)	Subgroup 4 (*n* = 201)	Subgroup 5 (*n* = 443)
Involvement with Pro Social Peers	Half of the peers	Half of the peers	A Few	Almost None	A Few
Parent Employment	Working now	Stay at Home	Working now	Working now	Working now
Parent Combined Income ($)	100 K–200 K	100 K–200 K	12 K–16 K	50 K–75 K	100 K–200 K
School Disengagement	Definitely not true	Definitely not true	Mostly True	Mostly not true	Definitely True
School Involvement	Mostly True	Mostly True	Mostly True	Mostly not true	Mostly Not true
Marital Status	Married	Married	Never Married	Married	Married
Social Role Engagement	Somewhat True	Somewhat True	Not True	Not True	Not true
Involvement with Rule Breaking Peers	Almost None	Almost None	Almost None	Almost None	Almost None
Likeness to Parent Behavior	Somewhat (like him)	Somewhat (like him)	Not like (him)	Not like (him)	Not like (him)

Demographic comparisons between FHSU-P subgroups and FHSU-N are shown in Table 3. Notable demographic difference between FHSU-P subgroups and the FHSU-N group, beyond what was observed between FHSU-P comparison with FHSU-N, emerged in sex such that Subgroup 5 showed significantly higher proportion of males (59.6%) compared to that in FHSU-N (51.5%; *χ*² = 13.41; *p* = 0.037; Table 3).

**Table 3:** Socio-demographic variables and SED indicators distribution by subgroups.

Demographic Details	FHSU-N (4,369)	FHSU-P (1,955)					Test statistics
		SG1 (744)	SG2 (300)	SG3 (267)	SG4 (201)	SG5 (443)	
Sex							
Male	2,250 (51.5%)	347 (46.64%)	141 (47%)	132 (49.44%)	98 (48.76%)	264 (59.59%)	SG1 (*χ*² = 7.82; *p* = 0.252) G2 (*χ*² = 6.30; *p* = 0.389) G3 (*χ*² = 1.02; *p* = 0.984) G4 (*χ*² = 1.04; *p* = 0.984) G5 (*χ*² = 13.41; *p* = 0.037)*
Female	2,110 (48.3%)	397 (53.36%)	158 (52.67%)	135 (50.56%)	103 (51.24%)	178 (40.18%)	
Other	9 (0.19%)	0 (0%)	1 (0.33%)	0 (0%)	0 (0%)	1 (0.33%)	
Parent age							
Parent Age	40.39 (6.67)	40.69 (6.64)	38.61 (6.22)	37.48 (8.64)	40.22 (7.29)	40.32 (6.34)	SG1 (t = 1.16; *p* = 0.246) G2 (t = −4.76; *p* < 0.001)* SG3 (t = −5.39; *p* < 0.001)* G4 (t = −0.318; *p* = 0.756) G5 (t = −0.210; *p* = 0.834)
Parent relationship							
Child's Biological Mother	3,810 (87.21%)	638 (85.75%)	267 (89%)	235 (88.01%)	174 (86.57%)	388 (87.58%)	SG1 (*χ*² = 50.46; *p* < 0.001)* G2 (*χ*² = 39.93; *p* < 0.001)* G3 (*χ*² = 53.28; *p* < 0.001)* G4 (*χ*² = 64.57; *p* < 0.001)* G5 (*χ*² = 71.80; *p* < 0.001)*
Child's Biological Father	485 (11.1%)	69 (9.27%)	15 (5%)	16 (5.99%)	13 (6.47%)	30 (6.77%)	
Adoptive Parent	20 (0.46%)	21 (2.82%)	6 (2%)	5 (1.87%)	10 (4.98%)	16 (3.61%)	
Child's Custodial Parent	18 (0.41%)	6 (0.81%)	6 (2%)	9 (3.37%)	1 (0.5%)	7 (1.58%)	
Other	36 (0.82)	10 (1.34%)	6 (2%)	2 (0.75%)	3 (1.49%)	2 (0.45%)	
Marital status							
Married	3,382 (77.41%)	553 (74.33%)	255 (85%)	47 (17.6%)	132 (65.67%)	323 (72.91%)	SG1 (*χ*² = 19.1; *p* < 0.001)* G2 (*χ*² = 29.55; *p* < 0.001)* G3 (*χ*² = 591.8; *p* < 0.001)* G4 (*χ*² = 17.92; *p* = 0.006)* G5 (*χ*² = 18.33; *p* = 0.005)*
Widowed	21 (0.59%)	5 (0.67%)	5 (1.67%)	4 (1.5%)	1 (0.5%)	4 (0.9%)	
Divorced	350 (8%)	72 (9.68%)	10 (3.33%)	45 (16.85%)	24 (11.94%)	55 (12.42%)	
Separated	117 (2.68%)	37 (4.97%)	12 (4%)	16 (5.99%)	10 (4.98%)	10 (2.26%)	
Never Married	330 (7.55%)	42 (5.65%)	7 (2.33%)	126 (47.19%)	21 (10.45%)	27 (6.09%)	
Living with Partner	155 (3.55%)	33 (4.44%)	10 (3.33%)	28 (10.49%)	13 (6.47%)	24 (5.42%)	
Refused to Answer	14 (0.32%)	2 (0.27%)	1 (0.33%)	1 (0.37%)	0 (0%)	0 (0%)	
Parent employment							
Working now (Full-Time or Part-Time)	3,164 (72.42%)	732 (37.44%)	0 (0%)	167 (8.54%)	154 (7.88%)	383 (19.59%)	SG1 (*χ*² = 278.26; *p* < 0.001) * G2 (*χ*² = 836.48; *p* < 0.001)* G3 (*χ*² = 14.07; *p* = 0.002)* G4 (*χ*² = 1.38; *p* = .2276) G5 (*χ*² = 44.46; *p* < 0.001)*
Combined income							
$100,000 through $199,999	1,513 (34.63%)	293 (14.99%)	81 (4.14%)	0 (0%)	52 (2.66%)	149 (7.62%)	SG1 (*χ*² = 15.12; *p* = 0.0001)* G2 (*χ*² = 4.87; *p* = 0.0235)* G3 (*χ*² = 135.85; *p* < 0.0001)* G4 (*χ*² = 4.46; *p* = .0.0325)* G5 (*χ*² = 0,05; *p* = 0.7934)*
Food availability							
Yes	4,162 (95.26%)	711 (95.56%)	283 (94.33%)	223 (83.52%)	184 (91.54%)	424 (95.71%)	SG1 (*χ*² = 2.91; *p* = 0.233) G2 (*χ*² = 0.66; *p* = 0.717) G3 (*χ*² = 69.59; *p* < 0.001)* G4 (*χ*² = 5.89; *p* = 0.052) G5 (*χ*² = 1.73; *p* = 0.420)
No	190 (4.35%)	33 (4.44%)	16 (5.33%)	42 (15.73%)	16 (7.96%)	19 (4.29%)	
Telephone services							
Yes	4,229 (96.8%)	737 (99.06%)	298 (99.33%)	236 (88.39%)	195 (97.01%)	438 (98.87%)	SG1 (*χ*² = 11.82; *p* = 0.003)* G2 (*χ*² = 0.61; *p* = 0.049) G3 (*χ*² = 53.80; *p* < 0.001)* G4 (*χ*² = 0.92; *p* = 0.633) G5 (*χ*² = 6.06; *p* = 0.048)
No	131 (3%)	7 (0.94%)	2 (0.67%)	31 (11.61%)	5 (2.49%)	5 (1.13%)	
Rent payment							
Yes	4,075 (93.27%)	682 (91.67%)	282 (94%)	200 (74.91%)	176 (87.56%)	412 (93%)	SG1 (*χ*² = 3.53; *p* = 0.169) G2 (*χ*² = 0.93; *p* = 0.628) G3 (*χ*² = 118.67; *p* < 0.001)* G4 (*χ*² = 11.09; *p* = 0.004) G5 (*χ*² = 1.40; *p* = 0.496)
No	282 (6.45%)	61 (8.2%)	18 (6%)	65 (24.34%)	23 (11.44%)	31 (7%)	
Eviction							
Yes	4,322 (98.92%)	742 (99.73%)	300 (100%)	260 (97.38%)	200 (99.5%)	442 (99.77%)	SG1 (*χ*² = 4.44; *p* = 0.108) G2 (*χ*² = 3.26; *p* = 0.196) G3 (*χ*² = 6.92; *p* < 0.031)* G4 (*χ*² = 0.68; *p* < 0.713) G5 (*χ*² = 2.97; *p* < 0.226)
No	42 (0.96%)	2 (0.27%)	0 (0%)	7 (2.62%)	1 (0.5%)	1 (0.23%)	
Utility payment							
Yes	4,228 (96.77%)	716 (96.24%)	297 (99%)	240 (89.89%)	195 (97.01%)	431 (97.29%)	SG1 (*χ*^2^ = 1.48; *p* = 0.478) SG2 (*χ*² = 4.68; *p* = 0.096) SG3 (*χ*² = 36.10; *p* < 0.001)* SG4 (*χ*² = 3.15; *p* = 0.206) SG5 (*χ*² = 1.26; *p* = 0.531)
No	137 (3.14%)	28 (3.76%)	3 (1%)	27 (10.11%)	5 (2.49%)	11 (2.48%)	
Healthcare expense							
Yes	4,226 (96.73%)	715 (96.1%)	285 (95%)	250 (93.63%)	189 (94.03%)	416 (93.91%)	SG1 (*χ*² = 1.94; *p* = 0.038) G2 (*χ*² = 3.33; *p* = 0.189) G3 (*χ*² = 8.30; *p* = 0.016)* G4 (*χ*² = 5.00; *p* = 0.820) G5 (*χ*² = 10.95; *p* = 0.004)*
No	138 (3.16%)	29 (3.9%)	15 (5%)	17 (6.37%)	12 (5.97%)	27 (6.09%)	
Dentalcare expenses							
Yes	4,062 (92.97%)	682 (91.67%)	267 (89%)	225 (84.27%)	180 (89.55%)	404 (91.2%)	SG1 (*χ*² = 3.715; *p* = 0.156) G2 (*χ*² = 8.01; *p* = 0.018) G3 (*χ*² = 29.85; *p* < 0.001)* G4 (*χ*² = 3.69; *p* = 0.158) G5 (*χ*² = 3.31; *p* = 0.191)
No	298 (6.82%)	62 (8.33%)	33 (11%)	42 (15.73%)	20 (9.95%)	39 (8.8%)	
Family history density of substance use problems							
FHD Score	0.10 (0.22)	0.56 (0.72)	0.59 (0.74)	0.88 (0.86)	0.73 (0.72)	0.63 (0.71)	SG1 (t = 17.24; *p* < 0.001)* G2 (t = 11.44; *p* < 0.001)* G3 (t = 14.73; *p* < 0.001)* G4 (t = 11.10; *p* < 0.001)* G5 (t = 15.63; *p* < 0.001)*

Regarding our sensitivity analysis examining subgroup differences in FHD scores, pairwise comparison between FHSU-P subgroups on FHD scores revealed that Subgroup 3 had the highest FHD scores compared to all other subgroup (t > −0.21, p_FDR_ < 0.0007; Supplementary Tables S6A,B). Moreover, Subgroup 1 and 5 did not differ on FHD scores (t = 0.013; p_FDR_ < 0.730; Supplementary Tables S6A,B).

#### Child behavior checklist (CBCL)

Comparison between the FHSU-P and FHSU-N participants revealed significantly higher scores in FHSU-P on internalizing (CE = 2.16, p_FDR_ < 0.0001), externalizing (CE = 2.56, p_FDR_ < 0.0001), and total (CE = 2.73, p_FDR_ < 0.0001) problem scores compared to FHSU-N participants.

Compared to the FHSU-N group, all five FHSU-P subgroups had significantly higher scores on internalizing (CE > 1.17, p_FDR_ < 0.0059), externalizing (CE > 1.27, p_FDR_ < 0.0014), and total problems (CE > 1.28, p_FDR_ < 0.0036), compared to the FHSU-N cohort. Among the FHSU-P subgroups, Subgroup 1 showed the lowest externalizing, internalizing, and total problems scores (Table 4; Supplementary Table S5).

**Table 4:** Comparison of UPPS-P impulsivity scores and CBCL symptom scores (internalizing, externalizing, and total) between FHSU-P subgroups, with statistically significant differences in the gradient highlighted (i.e., darker shades show greater difference).

CBCL and impulsivity measures	Subgroup 1 (*n* = 744)	Subgroup 2 (*n* = 300)	Subgroup 3 (*n* = 267)	Subgroup 4 (*n* = 201)	Subgroup 5 (*n* = 443)
Impulsive Behavior Scale—Negative Urgency	8.21 ± 2.57	8.52 ± 2.71	8.55 ± 2.77	8.77 ± 2.73	8.96 ± 2.6
Impulsive Behavior Scale—Lack of planning	7.47 ± 2.17	7.57 ± 2.26	7.48 ± 2.41	7.84 ± 2.24	8.81 ± 2.57
Impulsive Behavior Scale—Sensation Seeking	10.09 ± 2.61	9.7 ± 2.61	9.58 ± 2.77	9.28 ± 2.77	9.92 ± 2.53
Impulsive Behavior Scale—Positive Urgency	7.68 ± 2.92	7.75 ± 2.84	8.48 ± 2.95	8.17 ± 3.13	8.25 ± 3.03
Impulsive Behavior Scale—Lack of Perseverance	6.55 ± 1.9	6.8 ± 1.9	6.86 ± 2.14	7.56 ± 2.4	7.92 ± 2.32
Internal CBCL Syndrome	48.57 ± 10.3	50.63 ± 10.26	50.84 ± 10.85	51.03 ± 10.63	49.97 ± 10.56
External CBCL Syndrome	45.28 ± 9.73	47.34 ± 10.4	48.97 ± 10.75	47.87 ± 10.71	47.22 ± 9.84
Total Prob CBCL Syndrome	45.45 ± 10.67	47.78 ± 11.14	49.06 ± 11.62	49.24 ± 11.32	47.78 ± 10.66

			**Low**						**High**		

#### Impulsivity

Comparison between the FHSU-P and FHSU-N participants revealed significantly higher scores in FHSU-P on negative (CE = 0.17, p_FDR_ = 0.0276) and positive (CE = 0.25, p_FDR_ = 0.0012) urgency, and lack of planning (CE = 0.18, p_FDR_ = 0.0051) and perseverance (CE = 0.14, p_FDR_ = 0.0298), as summarized in Table 5. However, no significant between-group differences were observed in sensation seeking (CE = −0.0048, p_FDR_ = 0.9475).

**Table 5:** Comparison of mean and standard deviation of UPPS-P impulsivity scores and CBCL symptom scores (internalizing, externalizing, total) between FHSU-N and FHSU-P, with statistically significant differences highlighted by green shading.

CBCL and impulsivity measures	FHSU-N (*n* = 4,369)	FHSU-P (*n* = 1,955)
Impulsive Behavior Scale—Negative Urgency	8.34 ± 2.54	8.53 ± 2.66
Impulsive Behavior Scale—Lack of planning	7.66 ± 2.22	7.83 ± 2.38
Impulsive Behavior Scale—Sensation Seeking	9.87 ± 2.62	9.84 ± 2.64
Impulsive Behavior Scale—Positive Urgency	7.68 ± 2.81	7.98 ± 2.97
Impulsive Behavior Scale—Lack of Perseverance	6.9 ± 2.16	7.05 ± 2.16
Internal CBCL Syndrome	47.46 ± 10.06	49.77 ± 10.5
External CBCL Syndrome	44.06 ± 9.44	46.8 ± 10.18
Total Prob CBCL Syndrome	44.22 ± 10.42	47.22 ± 11.03

Pairwise comparisons between the FHSU-N and each FHSU-P subgroup suggested the presence of a gradient of impulsivity severity. Subgroup 1, which had risk-reducing traits (e.g., such as high parental support and low school disengagement), showed significantly lower scores on lack of perseverance (CE = −0.33, p_FDR_ = 0.0003). Subgroup 2 had comparable impulsivity scores as the FHSU-N group. Subgroup 3, with moderate traits, showed significantly higher scores on positive urgency (CE = 0.52, p_FDR_ = 0.0066). Subgroups 4 and 5 showed significantly higher impulsivity scores compared to FHSU-N. Subgroup 4 exhibited significantly higher scores on lack of perseverance (CE = 0.65, p_FDR_ = 0.0001) and higher sensation seeking (CE = 0.47, p_FDR_ = 0.0358), both compared to the FHSU-N group. Whereas Subgroup 5 showed significantly higher scores on negative urgency (CE = 0.58, p_FDR_ < 0.0001), positive urgency (CE = 0.53, p_FDR_ = 0.0003), lack of planning (CE = 1.10, p_FDR_ < 0.0001), and lack of perseverance (CE = 0.99, p_FDR_ < 0.00001).

In summary, increased scores in lack of perseverance was the most common finding for the FHSU-P Subgroups compared to FHSU-N controls. Also of interest is the notion of a gradient of increased impulsivity among the FHSU-P subgroups (Table 4; Supplementary Table S5) such that for Subgroup 2 there were no significantly increased impulsivity scores, Subgroups 1 and 3 showed increased impulsivity in only one domain, Subgroup 4 had increased impulsivity in 2 domains and Subgroup 5 in four domains (Supplementary Table S5). Additionally, the increase in impulsivity dimensions was not uniform across FHSU-P subgroups except for poor perseverance which was common to all but Subgroups 2 and 3.

#### Reward prediction errors

Comparison between the FHSU-P and FHSU-N participants revealed comparable PPE and NPE for both large reward and large loss (Large reward PPE: CE = 0.009, p_FDR_ = 0.8530; Large loss PPE: CE = −0.021, p_FDR_ = 0.7687; Large reward NPE: CE = 0.032, p_FDR_ = 0.2572; Large loss NPE: CE = −0.154, p_FDR_ = 0.0859].

Pairwise comparisons between the FHSU-N and each FHSU-P subgroup revealed no significant between-group differences (CE < 0.06, p_FDR_ > .44), except that Subgroup 1 showed less negative NPE to large losses compared to FHSU-N group (CE = −0.0178, p_FDR_ = .0301). Similarly, pairwise comparisons between the FHSU-P Subgroups revealed no significant differences in either NPE or PPE (CE < 0.11, p_FDR_ > .0611).

Overall, Subgroups 1 and 2 showed the lowest clinical symptoms and impulsivity, compared to the other FHSU-P subgroups, and Subgroup 5 showed the highest impulsivity traits. However, FHSU-P subgroups did not differ in RPE, except for Subgroup 1 which showed a greater large loss RPE compared to FHSU-N group.

Lastly, UPPS and CBCL scores were significantly correlated within each FHSU-P subgroup, PPE and NPE were not correlated with UPPS and CBCL measures ( Supplementary Figures S2A–F ).

## Discussion

In this study, we identified distinct subgroups of adolescents with familial risk for substance use disorder (SUD) based on sociodemographic and psychosocial factors. These subgroups fell along a spectrum of environmental advantage (i.e., higher family income, supportive parents, positive school and peer engagement) to disadvantage (e.g., lower income, unmarried parents, poor school and peer engagement) and differed markedly in both psychopathology symptoms and impulsivity levels. In particular, the more advantaged subgroups (Subgroups 1 and 2) showed lower psychopathology and impulsivity, whereas the more disadvantaged subgroups (Subgroups 3, 4, and 5) exhibited heightened psychopathology symptoms and elevated impulsivity patterns across multiple domains. Surprisingly, reward sensitivity did not differ between the subgroups, although this may reflect methodological constraints of the task used to derive prediction error estimates. Interestingly, within the subgroups with more favorable factors, Subgroup 2 was predominantly defined by the presence of a non-working, possibly stay-home parent, yet high family income, whereas Subgroup 1 was defined by working parents and a combination of additional favorable factors. Indeed, the impact of the role of stay-home vs. working parent on behavioral development is an active area of research, with some studies showing that children with stay-home mothers may exhibit less aggressive behaviors (68–70), while others showing no difference in behaviors for children with either working or stay-home parents (71). These findings highlight the importance of psychosocial context, individual differences in impulsivity, and potential methodological limitations in measuring reward processing within at-risk youth populations.

We also found Subgroups 1 and 2 to have the lowest levels of psychopathology symptoms as well as impulsivity in several domains within the FHSU-P subgroups, suggesting potential protective factors of intact parenting and parental relationships that may mitigate the impact of familial SUD risk (72). Conversely, Subgroups 3, 4, and 5 exhibited more pronounced psychopathology symptoms, suggesting possibly heightened vulnerability in those adolescents with disadvantageous psychosocial exposures and emphasizing the need for personalized approaches to assessment and intervention that account for individual differences in symptom presentation and risk profiles. However, note that unlike Subgroups 3 and 4, Subgroup 5 had greater proportion of females compared to males. These findings, especially of the aspects of impulsivity, are consistent with reports that implicate impulsivity in the etiology and maintenance of SUD (73, 74). Interestingly, however, such heightened vulnerability did not appear to be associated with the density of SUD problems in the family, since only Subgroup 3 showed the highest FHD scores compared to both more protective Subgroups 1 and 2, and more vulnerable Subgroups 4 and 5. Moreover, FHD scores were not significantly different between Subgroups 1 (most protective) and 5 (most vulnerable). From treatment planning standpoint, these findings underscore the importance of considering individual differences in impulsivity when assessing individuals with FHSU-P and designing targeted interventions aimed at reducing impulsive behaviors.

In contrast to our hypothesis, FHSU-P subgroups did not differ in terms of reward sensitivity, specifically quantified as computationally derived behavioral measures of positive and negative reward prediction error. Although it is plausible that these subgroups may indeed be comparable on positive and negative reward prediction error, we must also note that the MID task used in ABCD Study is not an optimal task to derive prediction error measures and for examining learning (75, 76). Perhaps the lack of differences in RPE between FHSU-P subgroups reflects this methodological limitation, and therefore, needs further validation. Nevertheless, recent reports have assessed the reliability of RPE, derived from the MID task, in encoding in young populations (48, 77), supporting the notion that RPE variable computed from the MID task may have utility in identifying specific patterns of reward-related brain activation, which in turn may also correlated with impulsivity measures in young populations. In case of the current study, comparing the groups on the RPE variable is an initial assessment for any possible reward sensitivity differences among the subgroups. Based on the established practices of utilizing computational variables in brain neuroimaging, RPE will need to be incorporated in an independent set of whole brain analyses of the sample to investigate for differences in brain activation and connectivity among the subgroups, which in turn may be linked to future drug initiation. An alternative consideration is that although impaired reward processing is associated with drug use initiation, it may not be related to FHSU-P or the underlying biology (78, 79) and in some cases overshadowed by complex interplay of other behavioral traits (80).

A major novelty of this study is that it attempts to subcategorize a large cohort of young FHSU-P youth in relation to environmental exposures in order to investigate two main objectives: first, that a set of relevant exposures will be able to differentiate independent subgroups with either favorable (possibly protective) and unfavorable environmental exposures, and second, that these groups may show significant differences in some behavioral (e.g., impulsivity) and clinical measures. As such we view the reported results as preliminary but strongly suggest that investigations in similar directions may benefit from using clustering techniques (e.g., k-means, as used in this study) or latent class analyses [latent profile analysis (LPA) or latent class growth analysis (LCGA)], to stratify individuals with FHSU into unique subgroups for more personalized assessments. Indeed, the reported significant differences in impulsivity scores appears to be the most interesting findings especially as a starting point of more detailed investigation for possible brain based differences among the subgroups as it is well established that there are correlations between high impulsivity and indexes of brain morphology and physiology (81, 82). In addition, as this relationship will need to be replicated, it offers the possibility to identify individuals with particular exposure profiles that may benefit from interventions that specifically target impulsivity subtypes (e.g., sensation seeking vs. negative urgency).

Indeed, these types of investigation may have a far-reaching implication for both early identification for high risk individuals as well as provide guidance for prevention and treatment of substance use in adolescence. Conceptually, findings of robust moderating effects from environmental exposures can inform multisectoral prevention and early intervention strategies that improve outcomes for at-risk youth. For example, information on the moderating effect of environmental factors may allow for promoting initiative that, on one hand, may support environmental factors with positive contribution to preventing substance use initiation in youth and, on the other, to modify environmental factors that may facilitate or predispose youth to early drug experimentation. Such initiatives may need to be based in family, school or neighborhood settings and include home based family therapy and group activities promoting problem solving skills (e.g., resisting peers' pressure for drug use, socializing without drug use etc.). Additionally, characterizing subgroups of youth who may have high levels of impulsive behaviors may allow clinicians to select most appropriate therapeutic strategies for addressing specific behaviors or symptoms. As impulsive behaviors may vary in respect to their inherent features (e.g., sensation seeking vs. positive or negative urgency) therapeutic goals may differ in reducing urges to seek novel experiences vs. managing urges to respond impulsively to different types of outcomes. Further, identifying impulsive behaviors as part of underlying disorders like ADHD may require considerations for pharmacological interventions in addition to behavioral therapies. In sum, more detailed information about stratifying large groups of youth who may have familial loading for SUD into “at risk” subgroups characterized by unique profiles of interacting environmental exposures and psychological features may allow for more effective “precision” approaches of preventing and treating adolescent substance use and related disorders.

Taken together, the next steps for this project are to conduct analyses of the longitudinal data that are available from ABCD and potentially determine which of the subgroups may exhibit high levels of substance initiation, at what ages these behaviors may occur and what types of biological characteristics (i.e., brain activation and connectivity) may be predictive of these behaviors. These types of analyses will be potentially able to produce results that can be used as evidence to support any recommendations for preventive, psychotherapeutic and psychopharmacological treatments for substance using adolescents as we have outlined in the introduction section. Additionally, such results may be applicable to recommendations for policy initiatives like age limits for legal substance use, parental consent for substance use treatment for minors, and legal consequences drug related offences for minors.

We want to also acknowledge relevant limitations that are important to consider when interpreting the findings. Firstly, the selection of demographic variables was guided by existing literature, but the findings here reinforce previous reports that advantageous and disadvantageous psychosocial exposures tend to cluster together along a positive-negative axis (27, 83, 84). Next, as discussed earlier, while the MID task is commonly used in research, its ability to fully capture prediction error or elucidate reinforcement learning mechanisms may be limited. Third, while we have done analysis of FHSU density, it was not included as factor for any analysis, that may have varying degree of resilience factors. As the next step, enhancing the study by incorporating additional measures, such as socio-demographic variables, social determinants of health, FHSU density or complementary tasks assessing reinforcement learning, would provide a more comprehensive understanding of the phenomena under investigation**.** Moreover, conducting similar clustering analyses in the FHSU-N group could help determine whether the identified subgroups reflect patterns specific to familial risk or instead capture broader socio-demographic or behavioral structures present across the general adolescent population.

## Conclusion

The findings of this study highlight the importance of psychosocial context in shaping the clinical presentation of adolescents with positive family history for substance use. Understanding these relationships is critical for informing targeted interventions aimed at mitigating the impact of familial risk factors on the development and maintenance of substance use disorders. Future research should further elucidate the mechanisms underlying these associations and explore novel intervention strategies tailored to individual risk profiles within the FHSU-P population.

## Data Availability

Publicly available datasets were analyzed in this study. This data can be found here: ABCD Study. https://abcdstudy.org/.
