# Supplementary material for: Environmentally derived subgroups of preadolescents with family history of substance use exhibit distinct patterns of psychopathology and reward-related behaviors: insights from the ABCD study
Source: Front Child Adolesc Psychiatry. 2025 Nov 13;4:1631474. doi: 10.3389/frcha.2025.1631474 (PMC12657496; doi:10.3389/frcha.2025.1631474)
Supplement: Supplementary file 1 [file Supplementaryfile1.docx]

**Supplement – FHSU Subgrouping – ABCD Study**


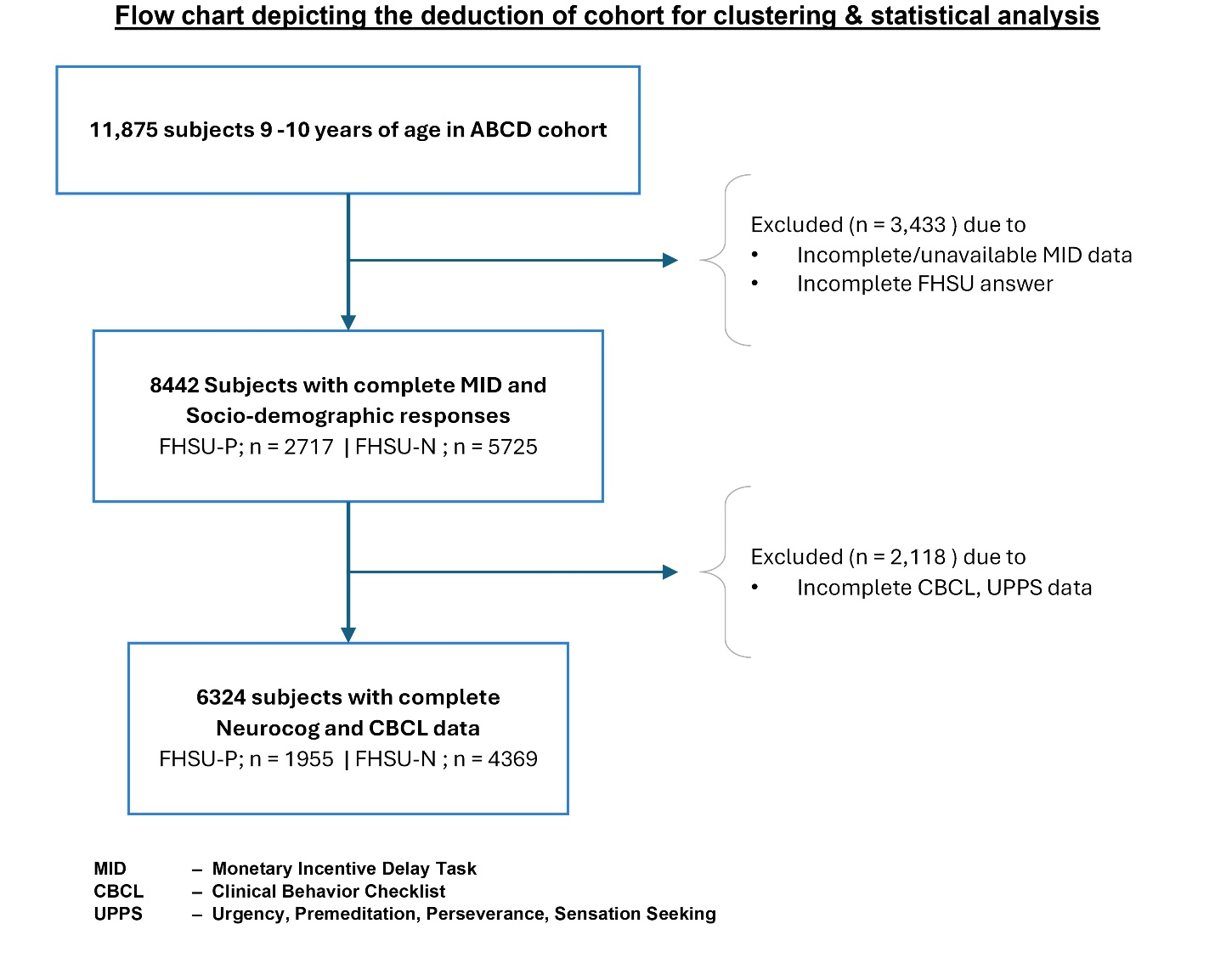


Figure S1: Flowchart depicting the deduction of cohort for statistical and clustering analysis

| Description | ABCD 5.0 (Field) | ABCD Details |
| --- | --- | --- |
| Negative Urgency | upps_y_ss_negative_urgency | UPPS-P for Children Short Form (ABCD-version), Negative Urgency: upps7_y + upps11_y + upps17_y + upps20_y; Validation: Minimum of three items answered |
| Lack of Planning | upps_y_ss_lack_of_planning | UPPS-P for Children Short Form (ABCD-version), Lack of Planning: upps6_y + upps16_y + upps23_y + upps28_y; Validation: Minimum of three items answered |
| Sensation Seeking | upps_y_ss_sensation_seeking | UPPS-P for Children Short Form (ABCD-version), Sensation Seeking: upps12_y + upps18_y + upps21_y + upps27_y; Validation: Minimum of three items answered |
| Positive Urgency | upps_y_ss_positive_urgency | UPPS-P for Children Short Form (ABCD-version), Positive Urgency: upps35_y + upps36_y + upps37_y + upps39_y; Validation: Minimum of three items answered |
| Lack of Perseverance | upps_y_ss_lack_of_perseverance | UPPS: Lack of Perseverance (GSSF) upps15_y plus upps19_y plus upps22_y plus upps24_y Validation : Minimum of three items answered |

Table S1: Impulsivity scores used for Statistical analysis.

| Description | ABCD 5.0 (Field) | ABCD Details |
| --- | --- | --- |
| Internal CBCL Syndrome | cbcl_scr_syn_internal_t | Internal CBCL Syndrome Scale (t-score) |
| External CBCL Syndrome | cbcl_scr_syn_external_t | External CBCL Syndrome Scale (t-score) |
| Total Prob CBCL Syndrome | cbcl_scr_syn_totprob_t | Total Probability CBCL Syndrome Scale (t-score) |

Table S2: CBCL scores used for Statistical analysis.

Variable used for Subgroup determination of FHSU-P

| Variables | ABCD - Questions | Response values | ABCD 5.0 (Field) |
| --- | --- | --- | --- |
| Sex | What sex was the child assigned at birth, on the original birth certificate? | 1 = Male  2 = Female  3 = Intersex-Male  4 = Intersex-Female  999 = Don't know  777 = Refuse to answer | demo_sex_v2, |
| Age | Age in months at the time of the Interview /test/ sampling / imaging | Number | demo_prnt_age_v2 |
| Race / Ethnicity (Child) | What race do you consider the child to be? Please check all that apply. | 1 = White; 2 = Black;  3 = Hispanic;  4 = Asian;  5 = Other | race_ethnicity |
| Child Grade | What grade is the child in? If it is summer, indicate grade starting in the fall? | 0 = KINDERGARTEN  1 = 1ST Grade  2 = 2ND Grade  3 = 3RD Grade  4 = 4TH Grade  5 = 5TH Grade  6 = 6TH Grade  7 = 7TH Grade  8 = 8TH Grade  9 = 9TH Grade  10 = 10TH Grade  11 = 11TH Grade  12 = 12TH Grade | demo_ed_v2 |
| Relationship with child | You are the | 1 = Child's Biological Mother 2 = Child's Biological Father 3 = Adoptive Parent 4 = Child's Custodial Parent 5 = Other | demo_prim |
| Partnership (living with/not living with) | Are you now married, widowed, divorced, separated, never married or living with a partner? | 1 = Married 2 = Widowed 3 = Divorced 4 = Separated 5 = Never married 6 = Living with partner 777 = Refused to answer | demo_prnt_marital_v2 |
| Employment (Yes/No) | Are you working now, looking for work, retired, stay at home parent, a student, or something else? | 1 = Working now: Full Time/Part Time 2 = Temporarily Laid off 9 = Sick Leave 10 = Maternity Leave 3 = Looking for work 11 = Unemployed not looking for work 4 = Retired 5 = Disabled: Permanently or Temporarily 6 = Stay at Home Parent 7 = Student 8 = Other (Specify): 777 = Refused to answer | demo_prnt_empl_v2 |
| Psychiatric History: Parental Help-Seeking (recoded 0=neither parent, 1= one parent, 2=both parents) | Has ANY blood relative of your child ever been to a doctor or a counselor about any emotional or mental problems, or problems with alcohol or drugs? | 1 = Yes 0 = No 999 = Don't know 777 = Refuse to answer | fam_history_11_yes_no |
| Substance use history | Has ANY blood relative of your child ever had any problems due to drugs, such as: Marital separation or divorce; Laid off or fired from work; Arrests or DUIs; Drugs harmed their health; In a drug treatment program; Suspended or expelled from school 2 or more times; Isolated self from family, caused arguments or were high a lot. | 2 = Yes 0 = No 999 = Don't know 777 = Refuse to answer | fam_history_5_yes_no |
| Family Income | What is your TOTAL COMBINED FAMILY INCOME for the past 12 months? This should include income (before taxes and deductions) from all sources, wages, rent from properties, social security, disability and/or veteran's benefits, unemployment benefits, workman's compensation, help from relative (include child payments and alimony), and so on | 1 = Less than $5,000 2 = $5,000 through $11,999 3 = $12,000 through $15,999 4 = $16,000 through $24,999 5 = $25,000 through $34,999 6 = $35,000 through $49,999 7 = $50,000 through $74,999 8 = $75,000 through $99,999 9 = $100,000 through $199,999 10 = $200,000 and greater 999 = Don't know 777 = Refuse to answer | demo_comb_income_v2 |
| Severe financial adversity – Food Availability | In the past 12 months, has there been a time when you and your immediate family experienced any of the following: Needed food but couldn't afford to buy it or couldn't afford to go out to get it? | 0 = No ; 1 = Yes ;  777 = Refuse to answer | demo_fam_exp1_v2, |
| Severe financial adversity – Telephone services | In the past 12 months, has there been a time when you and your immediate family experienced any of the following: Were without telephone service because you could not afford it? | 0 = No ; 1 = Yes ;  777 = Refuse to answer | demo_fam_exp2_v2, |
| Severe financial adversity – Rent Payment | In the past 12 months, has there been a time when you and your immediate family experienced any of the following: Didn't pay the full amount of the rent or mortgage because you could not afford it? | 0 = No ; 1 = Yes ;  777 = Refuse to answer | demo_fam_exp3_v2, |
| Severe financial adversity - Eviction | In the past 12 months, has there been a time when you and your immediate family experienced any of the following: Were evicted from your home for not paying the rent or mortgage? | 0 = No ; 1 = Yes ;  777 = Refuse to answer | demo_fam_exp4_v2, |
| Severe financial adversity – Utility payment | In the past 12 months, has there been a time when you and your immediate family experienced any of the following: Had services turned off by the gas or electric company, or the oil company wouldn't deliver oil because payments were not made? | 0 = No ; 1 = Yes ;  777 = Refuse to answer | demo_fam_exp5_v2, |
| Severe financial adversity – Healthcare expense | In the past 12 months, has there been a time when you and your immediate family experienced any of the following: Had someone who needed to see a doctor or go to the hospital but didn't go because you could not afford it? | 0 = No ; 1 = Yes ;  777 = Refuse to answer | demo_fam_exp6_v2, |
| Severe financial adversity – Dentalcare expenses | In the past 12 months, has there been a time when you and your immediate family experienced any of the following: Had someone who needed a dentist but couldn't go because you could not afford it? | 0 = No ; 1 = Yes ;  777 = Refuse to answer | demo_fam_exp7_v2, |
| Acceptance Subscale- CRPBI (Child's Report of Parental Behavior Inventory)- Acceptance Subscale: Mean of Report by Parent: | CRPBI - Acceptance Subscale Mean of Report by Parent Completing Protocol by youth:  First caregiver (caregiver participating in study/completing protocol).  (Makes me feel better after talking over my worries with him/her + Smiles at me very often + Is able to make me feel better when I am upset + Believes in showing his/her love for me + Is easy to talk to.) | 1 = Not like him/her;  2 = Somewhat like him/her;  3 = A lot like him/her | crpbi_y_ss_parent  (crpbi_parent1_y + crpbi_parent2_y + crpbi_parent3_y], crpbi_parent4_y + crpbi_parent5_y)/5;  Validation: Minimum of 4 items answered |
| Parental Monitoring (Calculated) | Parental Monitoring Summary Score: Mean of all items  (How often do your parents/guardians know where you are? **+** How often do your parents know who you are with when you are not at school and away from home? **+** If you are at home when your parents or guardians are not, how often do you know how to get in touch with them? **+** How often do you talk to your parent or guardian about your plans for the coming day, such as your plans about what will happen at school or what you are going to do with friends**? +** In an average week, how many times do you and your parents/guardians eat dinner together? | 1 = Yes = completely 2 = Yes = quite well 3 = Yes = partly 4 = No = very little 5 = No = nothing 777 = Decline to answer | parental_monitor_ss_mean  Mean (parent_monitor_q1_y + parent_monitor_q2_y + parent_monitor_q3_y + parent_monitor_q4_y parent_monitor_q5_y)/5; Validation: no minimum number of answers - |
| Overall school performance, grades, drop in grades, in-school emotional support, in-school learning support, in-school aid | In the past year or past several months, has there been a drop in your child's grades? | 1 = Yes ;  2 = No ;  -1 = Not applicable /No aplica | kbi_p_c_drop_in_grades |
| Friendship (KSADS Background) | Does your child have a best friend? | 1 = Yes ;  2 = No ;  3 = Not sure ;  777 = Decline to answer | kbi_p_c_best_friend |
| Friendship Longitudinal; (KSADS Background) | Since we last saw you, …  Does your child have a best friend? | 1 = Yes ;  2 = No ;  3 = Not sure ;  777 = Decline to answer | kbi_p_c_best_friend_l |
| School and Neighborhood Bullying Longitudinal (KSADS Background) | Does your child have any problems with bullying at school or in your neighborhood? | 1 = Yes ;  2 = No ;  777 = Decline to answer | kbi_p_c_bully |
| School and Neighborhood Bullying (KSADS Background) | Since we last saw you, did your child have any problems with bullying at school or in your neighborhood? | 1 = Yes ;  2 = No ;  777 = Decline to answer | kbi_p_c_bully_l |
| School and Neighborhood Bullying (KSADS Background) | Do you have any problems with bullying at school or in your neighborhood? | 1 = Yes;  0 = No | ksads_bully_raw_26 |
| School Risk and Protective Factors (Disengagement, Involvement) (SRPF) | School disengagement Subscale,  Sum: (Usually, school bores me + Getting good grades is not so important to me) | 1 = NO!;  2 = no;  3 = yes;  4 = YES! / /Mark (the BIG) YES! if you think the statement is definitely true for you. Mark (the little) yes if you think the statement is mostly true for you. Mark (the little) no if you think the statement is mostly not true for you. Mark (the BIG) NO! if you think the statement is definitely not true for you | srpf_y_ss_dfs  sum (school_15_y + school_17_y ) |
| School Risk and Protective Factors (Disengagement, Involvement) (SRPF) | SRPF School Involvement Subscale,  Sum: (I like school because I do well in class + I feel I'm just as smart as other kids my age + There are lots of chances to be part of class discussions or activities + In general, I like school a lot.); | 1 = NO!;  2 = no;  3 = yes;  4 = YES! / /Mark (the BIG) YES! if you think the statement is definitely true for you. Mark (the little) yes if you think the statement is mostly true for you. Mark (the little) no if you think the statement is mostly not true for you. Mark (the BIG) NO! if you think the statement is definitely not true for you | srpf_y_ss_iiss sum (school_8_y + school_9_y + school_10_y + school_12_y )  Validation: Minimum of three items answered |
| School Risk and Protective Factors (School Environment) (SRPF) | SRPF School Environment Subscale,  Sum: (In my school, students have lots of chances to help decide things like class activities and rules + I get along with my teachers. + My teacher(s) notices when I am doing a good job and lets me know about it. + There are lots of chances for students in my school to get involved in sports, clubs, or other school activities outside of class + I feel safe at my school + The school lets my parents know when I have done something well. | 1 = NO!;  2 = no;  3 = yes;  4 = YES!   //Mark (the BIG) YES! if you think the statement is definitely true for you. Mark (the little) yes if you think the statement is mostly true for you. Mark (the little) no if you think the statement is mostly not true for you. Mark (the BIG) NO! if you think the statement is definitely not true for you | srpf_y_ss_ses  Sum: school_2_y + school_3_y + school_4_y + school_5_y + school_6_y + school_7_y; Validation: Minimum of five items answered |
| Peer Network Health: Protective Scale Score (social support by peers) | Peer Network Health: Protective Scale Score;  sum (During the last 6 months, have any of your close friends ever suggested that you not use drugs or alcohol? + During the last 6 months, have any of your close friends given you help with school, with money, with transportation, or help by talking through problems? + How much help did your close friends give you? Pick a number between 1 and 10. + During the last 6 months, have any of your close friends encouraged you to get or stay involved with sports/exercise, school teams or clubs, volunteering, or religious activities? + How much did your close friends encourage you? Pick a number between 1 and 10. ) | 0=No; 3=Yes   During the last 6 months, have any of your close friends ever suggested that you not use drugs or alcohol? | pnh_ss_protective_scale  (pnh_substance, pnh_help, pnh_how_much_help, pnh_encourage, pnh_how_much_encourage);  Validation: Items #1,#2, and #4 must be answered |
| Involvement with Prosocial Peers | Involvement with Prosocial peers  Have your Peers (Are athletes + Go to church once a month or more often + Are excellent students (GPA 3.5 [B+] or higher)) | 1=None or almost none;  2=A few;  3=Half;  4=Most;  5=All or almost all; 999=Don't know | pbp_ss_prosocial_peers  sum (pbp_athletes, pbp_church + pbp_good_student)  Validation: Minimum of two items must be answered |
| Involvement with Rule Breaking / Delinquent Peers | Involvement with Rule Breaking/Delinquent peers  Have your Peers (Have skipped school + Have been suspended from school + Have shoplifted occasionally ) | 1=None or almost none; 2=A few;  3=Half;  4=Most;  5=All or almost all; 999=Don't know | pbp_ss_rule_break  sum  (pbp_skip_school, pbp_suspended, pbp_shop_lifted)  Validation: Minimum of two items must be answered |
| Social Role and Engagement | Prosocial Behavior Subscale: Mean of Youth Self Report:  (I try to be nice to other people. I care about their feelings + I am helpful if someone is hurt, upset, or feeling sick +I often offer to help others (parents, teachers, children) | 0 = Not True;  1 = Somewhat True;  2 = Certainly True | psb_y_ss_mean  ( prosocial_q1_y + prosocial_q2_y + prosocial_q2_y ) /3  Validation: Minimum of two items answered |
| Discrimination Questionnaire (total score) | Discrimination Total score: Mean of Discrimination measures  (How often do the following people treat you unfairly or negatively because of your ethnic background? Teachers: + How often do the following people treat you unfairly or negatively because of your ethnic background? Other adults outside school: + How often do the following people treat you unfairly or negatively because of your ethnic background? Other students: + I feel that others behave in an unfair or negative way toward my ethnic group + I feel that I am not wanted in American society + I don't feel accepted by other Americans + I feel that other Americans have something against me) | 1 = almost never  2 = rarely  3 = sometimes  4 = often  5 = very often  777 = don't know  999 = refused to answer | dim_y_ss_mean  mean (dim_matrix_q1,dim_matrix_q2,dim_matrix_q3,dim_matrix_q4 ,dim_matrix_q5,dim_matrix_q6,dim_matrix_q7);  Validation  Only add if value 1 - 5;  Minimum of four items must be answered |
| Youth Substance Use | Have you ever tried at any time in your life? A sip of alcohol such as beer, wine or liquor (rum, vodka, gin, whiskey) | 0 = No;  1 = Yes | tlfb_alc_sip |

Table S3: Socio-demographic, SES and Mean score variables used for Cluster determination.

| Clusters (N) | Silhouette score |
| --- | --- |
| 2 | 0.16974459749597592 |
| 3 | 0.15136249781543207 |
| 4 | 0.15736254408366787 |
| 5 | 0.1536565945838913 |
| 6 | 0.11974453116020221 |
| 7 | 0.1168287336302872 |
| 8 | 0.11196196966992322 |
| 9 | 0.11512060535904438 |

Table S4: Cluster determination and Silhouette scores

| Measures | Subgroup | | Beta | | SE | | P(fdr) | | Lower CI | | Upper CI | | Z | | Hazard  Ratio |
| --- | --- | --- | --- | --- | --- | --- | --- | --- | --- | --- | --- | --- | --- | --- | --- |
| Lack of Perseverance | Subgroup 1 | | -0.3323 | | 0.0854 | | 0.0003 | | -0.4998 | | -0.1649 | | -3.8898 | | 0.7172 |
|  | Subgroup 2 | | -0.1271 | | 0.1295 | | 0.4899 | | -0.3809 | | 0.1268 | | -0.9811 | | 0.8807 |
|  | Subgroup 3 | | 0.0064 | | 0.1377 | | 0.9631 | | -0.2635 | | 0.2763 | | 0.0463 | | 1.0064 |
|  | Subgroup 4 | | 0.6500 | | 0.1544 | | 0.0001 | | 0.3474 | | 0.9526 | | 4.2100 | | 1.9156 |
|  | Subgroup 5 | | 0.9976 | | 0.1070 | | 0.0000 | | 0.7878 | | 1.2073 | | 9.3215 | | 2.7116 |
| Lack of planning | Subgroup 1 | | -0.1685 | | 0.0891 | | 0.1410 | | -0.3431 | | 0.0062 | | -1.8905 | | 0.8450 |
|  | Subgroup 2 | | -0.0633 | | 0.1349 | | 0.7677 | | -0.3278 | | 0.2012 | | -0.4691 | | 0.9387 |
|  | Subgroup 3 | | -0.0641 | | 0.1438 | | 0.7677 | | -0.3459 | | 0.2178 | | -0.4455 | | 0.9380 |
|  | Subgroup 4 | | 0.2009 | | 0.1618 | | 0.3680 | | -0.1164 | | 0.5181 | | 1.2410 | | 1.2224 |
|  | Subgroup 5 | | 1.1045 | | 0.1120 | | 0.0001 | | 0.8850 | | 1.3240 | | 9.8609 | | 3.0177 |
| Negative Urgency | Subgroup 1 | | -0.1297 | | 0.1026 | | 0.2752 | | -0.3308 | | 0.0714 | | -1.2637 | | 0.8784 |
|  | Subgroup 2 | | 0.2119 | | 0.1556 | | 0.2598 | | -0.0930 | | 0.5168 | | 1.3621 | | 1.2360 |
|  | Subgroup 3 | | 0.1350 | | 0.1654 | | 0.4890 | | -0.1893 | | 0.4592 | | 0.8158 | | 1.1445 |
|  | Subgroup 4 | | 0.3676 | | 0.1854 | | 0.0814 | | 0.0042 | | 0.7311 | | 1.9825 | | 1.4443 |
|  | Subgroup 5 | | 0.5825 | | 0.1285 | | 0.0000 | | 0.3305 | | 0.8344 | | 4.5315 | | 1.7905 |
| Positive Urgency | Subgroup 1 | | 0.0185 | | 0.1133 | | 0.8702 | | -0.2035 | | 0.2405 | | 0.1635 | | 1.0187 |
|  | Subgroup 2 | | 0.1087 | | 0.1717 | | 0.5746 | | -0.2278 | | 0.4453 | | 0.6331 | | 1.1148 |
|  | Subgroup 3 | | 0.5204 | | 0.1828 | | 0.0066 | | 0.1622 | | 0.8787 | | 2.8471 | | 1.6828 |
|  | Subgroup 4 | | 0.4059 | | 0.2051 | | 0.0637 | | 0.0040 | | 0.8079 | | 1.9794 | | 1.5007 |
|  | Subgroup 5 | | 0.5348 | | 0.1421 | | 0.0003 | | 0.2564 | | 0.8133 | | 3.7644 | | 1.7071 |
| Sensation Seeking | Subgroup 1 | | 0.2336 | | 0.1039 | | 0.0520 | | 0.0299 | | 0.4374 | | 2.2478 | | 1.2632 |
|  | Subgroup 2 | | -0.1356 | | 0.1577 | | 0.4678 | | -0.4448 | | 0.1735 | | -0.8600 | | 0.8732 |
|  | Subgroup 3 | | -0.1783 | | 0.1676 | | 0.3833 | | -0.5067 | | 0.1502 | | -1.0638 | | 0.8367 |
|  | Subgroup 4 | | -0.4707 | | 0.1872 | | 0.0358 | | -0.8376 | | -0.1038 | | -2.5147 | | 0.6245 |
|  | Subgroup 5 | | -0.0071 | | 0.1299 | | 0.9563 | | -0.2618 | | 0.2475 | | -0.0549 | | 0.9929 |
| Externalizing Syndrome | Subgroup 1 | 1.2760 | | 0.3848 | | 0.0014 | | 0.5218 | | 2.0301 | | 3.3162 | | 3.5821 | |
|  | Subgroup 2 | 2.8238 | | 0.5855 | | 0.0000 | | 1.6763 | | 3.9713 | | 4.8233 | | 16.8408 | |
|  | Subgroup 3 | 4.2945 | | 0.6207 | | 0.0000 | | 3.0780 | | 5.5110 | | 6.9190 | | 73.2950 | |
|  | Subgroup 4 | 3.5521 | | 0.6827 | | 0.0000 | | 2.2141 | | 4.8901 | | 5.2032 | | 34.8868 | |
|  | Subgroup 5 | 3.0653 | | 0.4766 | | 0.0000 | | 2.1311 | | 3.9995 | | 6.4310 | | 21.4412 | |
| Internalizing CBCL Syndrome | Subgroup 1 | 1.1701 | | 0.4053 | | 0.0059 | | 0.3757 | | 1.9646 | | 2.8870 | | 3.2225 | |
|  | Subgroup 2 | 2.5213 | | 0.6170 | | 0.0001 | | 1.3120 | | 3.7306 | | 4.0865 | | 12.4452 | |
|  | Subgroup 3 | 3.5050 | | 0.6541 | | 0.0000 | | 2.2229 | | 4.7871 | | 5.3583 | | 33.2811 | |
|  | Subgroup 4 | 3.4484 | | 0.7174 | | 0.0000 | | 2.0423 | | 4.8545 | | 4.8069 | | 31.4498 | |
|  | Subgroup 5 | 2.1967 | | 0.5014 | | 0.0000 | | 1.2139 | | 3.1795 | | 4.3809 | | 8.9955 | |
| Total Prob CBCL Syndrome | Subgroup 1 | 1.2896 | | 0.4193 | | 0.0036 | | 0.4678 | | 2.1113 | | 3.0759 | | 3.6313 | |
|  | Subgroup 2 | 2.7829 | | 0.6392 | | 0.0000 | | 1.5300 | | 4.0359 | | 4.3535 | | 16.1665 | |
|  | Subgroup 3 | 4.4383 | | 0.6782 | | 0.0000 | | 3.1091 | | 5.7675 | | 6.5446 | | 84.6310 | |
|  | Subgroup 4 | 4.5451 | | 0.7318 | | 0.0000 | | 3.1108 | | 5.9794 | | 6.2110 | | 94.1717 | |
|  | Subgroup 5 | 3.3082 | | 0.5148 | | 0.0000 | | 2.2993 | | 4.3172 | | 6.4268 | | 27.3367 | |
| Large Reward Positive PE | Subgroup 1 | -0.0440 | | 0.0334 | | 0.9146 | | -0.1094 | | 0.0214 | | -1.3176 | | 0.9570 | |
|  | Subgroup 2 | 0.0040 | | 0.0222 | | 0.4461 | | -0.0394 | | 0.0475 | | 0.1822 | | 1.0040 | |
|  | Subgroup 3 | 0.0489 | | 0.0402 | | 0.8117 | | -0.0298 | | 0.1277 | | 1.2185 | | 1.0502 | |
|  | Subgroup 4 | -0.0218 | | 0.0356 | | 0.4461 | | -0.0915 | | 0.0480 | | -0.6111 | | 0.9785 | |
|  | Subgroup 5 | 0.0357 | | 0.0278 | | 0.4461 | | -0.0188 | | 0.0902 | | 1.2848 | | 1.0364 | |
| Large Loss Positive PE | Subgroup 1 | 0.0347 | | 0.0339 | | 0.7120 | | -0.0318 | | 0.1012 | | 1.0225 | | 1.0353 | |
|  | Subgroup 2 | -0.0238 | | 0.0226 | | 0.7120 | | -0.0680 | | 0.0205 | | -1.0529 | | 0.9765 | |
|  | Subgroup 3 | -0.0592 | | 0.0409 | | 0.8406 | | -0.1395 | | 0.0210 | | -1.4472 | | 0.9425 | |
|  | Subgroup 4 | 0.0174 | | 0.0362 | | 0.6036 | | -0.0536 | | 0.0884 | | 0.4811 | | 1.0176 | |
|  | Subgroup 5 | -0.0036 | | 0.0283 | | 0.9791 | | -0.0592 | | 0.0519 | | -0.1283 | | 0.9964 | |
| Large Reward Negative PE | Subgroup 1 | 0.0103 | | 0.0426 | | 0.6059 | | -0.0733 | | 0.0938 | | 0.2408 | | 1.0103 | |
|  | Subgroup 2 | 0.0302 | | 0.0282 | | 0.9717 | | -0.0251 | | 0.0856 | | 1.0701 | | 1.0307 | |
|  | Subgroup 3 | 0.0510 | | 0.0514 | | 0.6059 | | -0.0497 | | 0.1517 | | 0.9931 | | 1.0523 | |
|  | Subgroup 4 | 0.0484 | | 0.0455 | | 0.6059 | | -0.0407 | | 0.1376 | | 1.0645 | | 1.0496 | |
|  | Subgroup 5 | 0.0330 | | 0.0355 | | 0.6059 | | -0.0366 | | 0.1026 | | 0.9280 | | 1.0335 | |
| Large Loss Negative PE | Subgroup 1 | -0.0178 | | 0.0395 | | 0.0302 | | -0.0952 | | 0.0595 | | -0.4520 | | 0.9823 | |
|  | Subgroup 2 | -0.0735 | | 0.0262 | | 0.8863 | | -0.1249 | | -0.0222 | | -2.8065 | | 0.9291 | |
|  | Subgroup 3 | -0.0373 | | 0.0476 | | 0.8863 | | -0.1306 | | 0.0561 | | -0.7826 | | 0.9634 | |
|  | Subgroup 4 | 0.0183 | | 0.0422 | | 0.8678 | | -0.0643 | | 0.1009 | | 0.4335 | | 1.0184 | |
|  | Subgroup 5 | -0.0286 | | 0.0329 | | 0.8678 | | -0.0931 | | 0.0360 | | -0.8675 | | 0.9718 | |

Table S5: Mixed Linear Model outcomes. FHSU-P Subgroup comparisons with FHSU-N

| **P-value** | Subgroup 1 | Subgroup 2 | Subgroup 3 | Subgroup 4 | Subgroup 5 | FHSU-N |
| --- | --- | --- | --- | --- | --- | --- |
| Subgroup 1 | - | 0.5231 | 0.0000 | 0.5231 | 0.7300 | 0.0000 |
| Subgroup 2 | 0.5231 | - | 0.0000 | 0.3248 | 0.4257 | 0.0000 |
| Subgroup 3 | 0.0000 | 0.0000 | - | 0.0007 | 0.0000 | 0.0000 |
| Subgroup 4 | 0.5231 | 0.3248 | 0.0007 | - | 0.6343 | 0.0000 |
| Subgroup 5 | 0.7300 | 0.4257 | 0.0000 | 0.6343 | - | 0.0000 |
| FHSU-N | 0.0000 | 0.0000 | 0.0000 | 0.0000 | 0.0000 | - |

Table S6A: P-values (FDR corrected) for between-subgroup (FHSU-P) comparisons on family history density of SUD (FHD). Statistically significant values are highlighted in purple.

| **Estimate** | Subgroup 1 | Subgroup 2 | Subgroup 3 | Subgroup 4 | Subgroup 5 | FHSU-N |
| --- | --- | --- | --- | --- | --- | --- |
| Subgroup 1 | - | -0.0418 | 0.2433 | 0.0382 | 0.0134 | 0.4860 |
| Subgroup 2 | -0.0418 | - | 0.2851 | 0.0800 | 0.0552 | 0.4692 |
| Subgroup 3 | 0.2433 | 0.2851 | - | -0.2052 | -0.2300 | 0.7312 |
| Subgroup 4 | 0.0382 | 0.0800 | -0.2052 | - | -0.0248 | 0.5409 |
| Subgroup 5 | 0.0134 | 0.0552 | -0.2300 | -0.0248 | - | 0.5208 |
| FHSU-N | 0.4860 | 0.4692 | 0.7312 | 0.5409 | 0.5208 |  |

Table S6B: Estimates of between-subgroup (FHSU-P) comparisons on family history density of SUD (FHD).

**Sub-Group SG1**

Total number of family members with FHSU density - 487


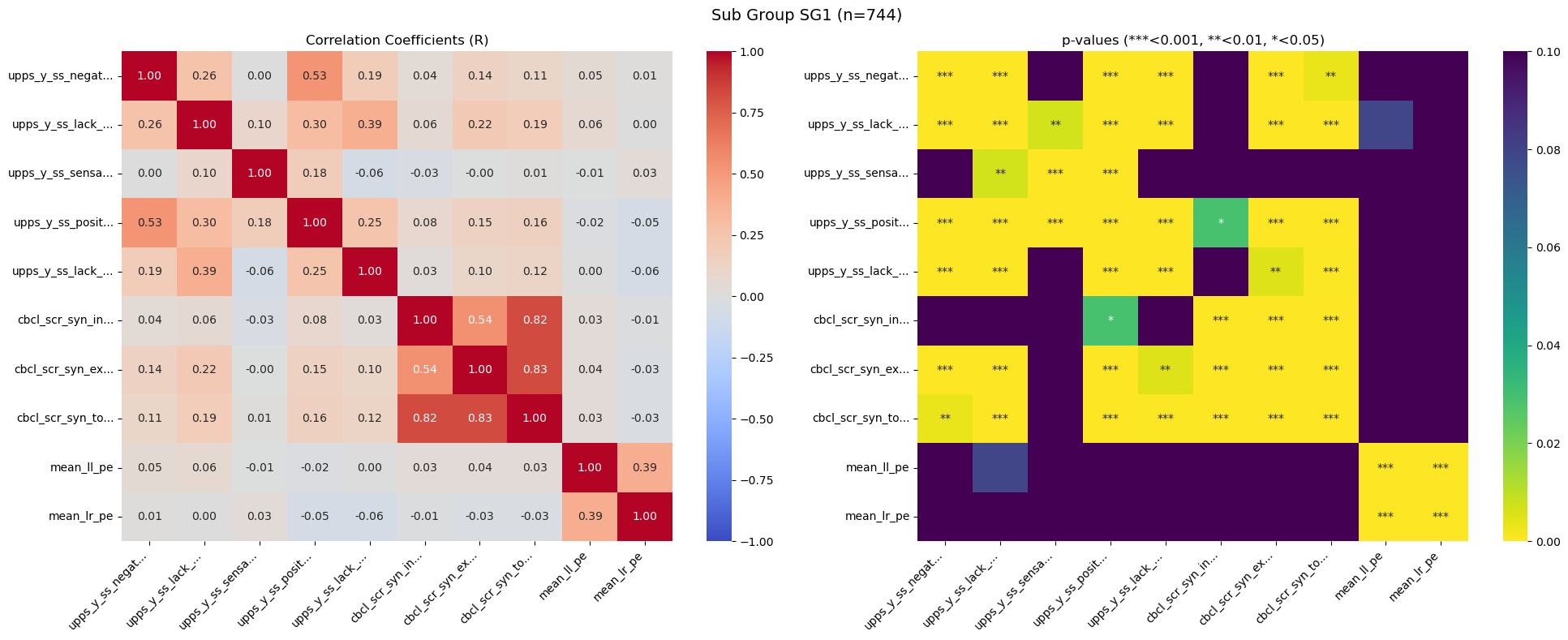


Figure S2A: Correlation between CBCL, UPPS and RPE variables for Subgroup 1

**Sub-Group SG2**

Total number of family members with FHSU density - 191


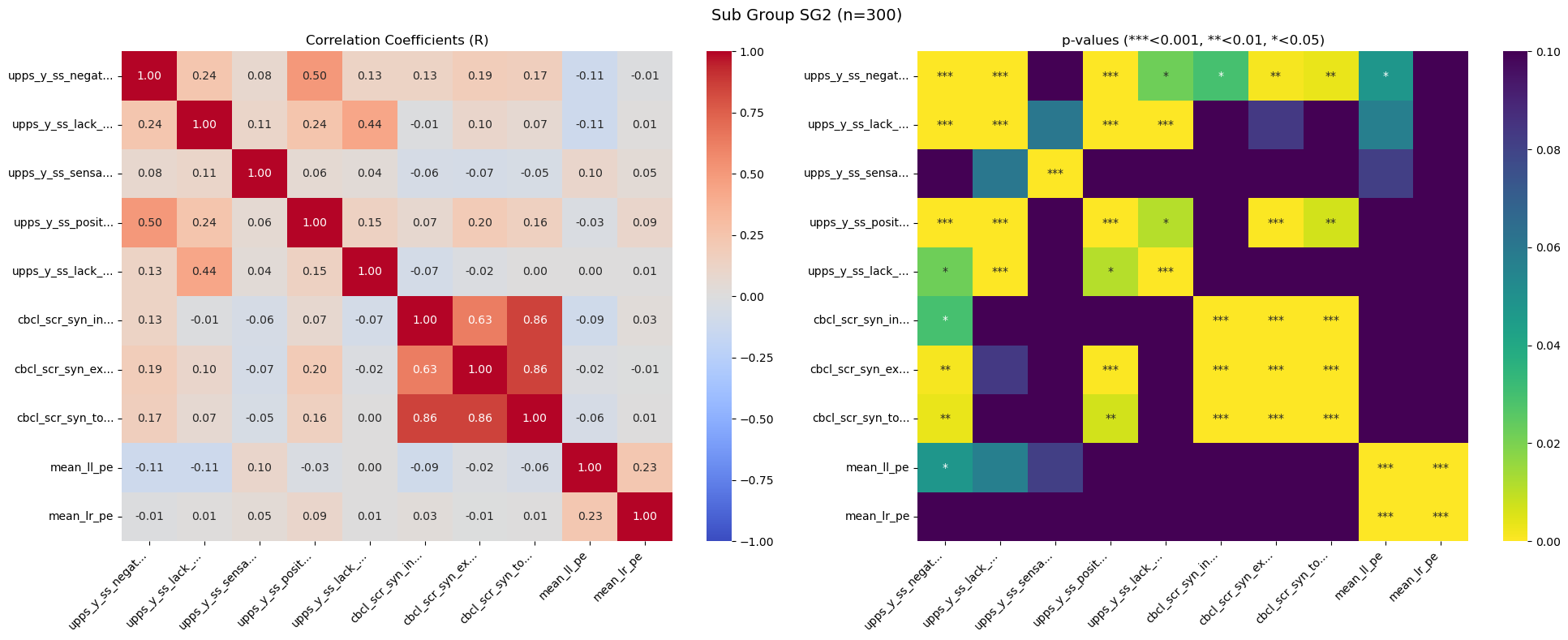


Figure S2B: Correlation between CBCL, UPPS and RPE variables for Subgroup 2

**Sub-Group SG3**

Total number of family members with FHSU density - 214


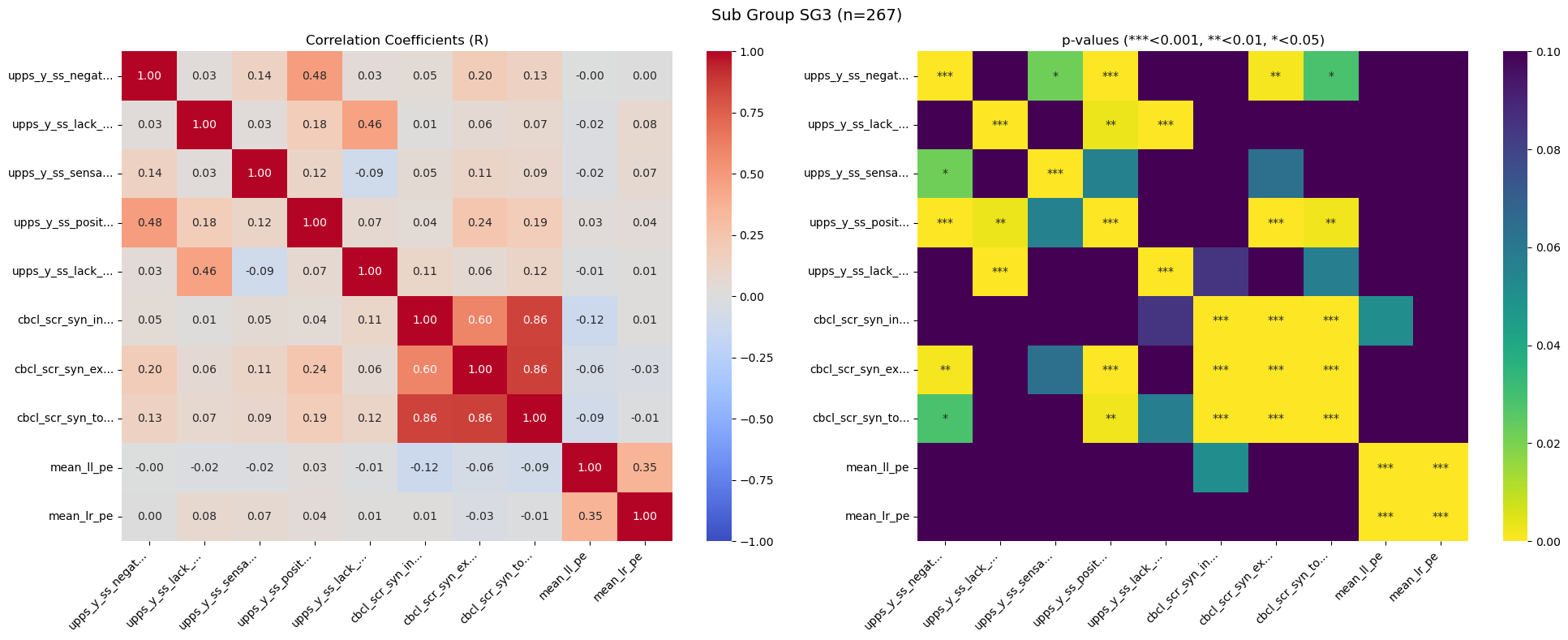


Figure S2C: Correlation between CBCL, UPPS and RPE variables for Subgroup 3

**Sub-Group SG4**

Total number of family members with FHSU density - 150


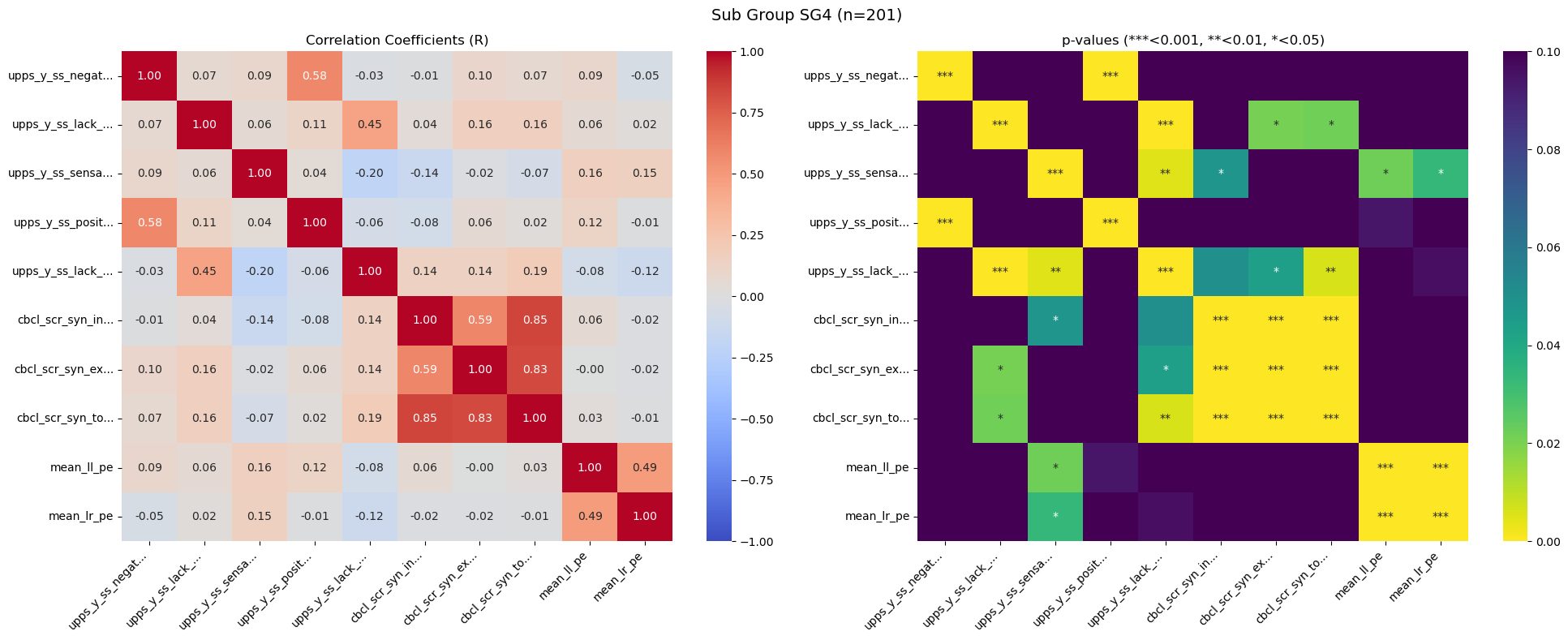


Figure S2D: Correlation between CBCL, UPPS and RPE variables for Subgroup 4

**Sub-Group SG5**

Total number of family members with FHSU density - 304


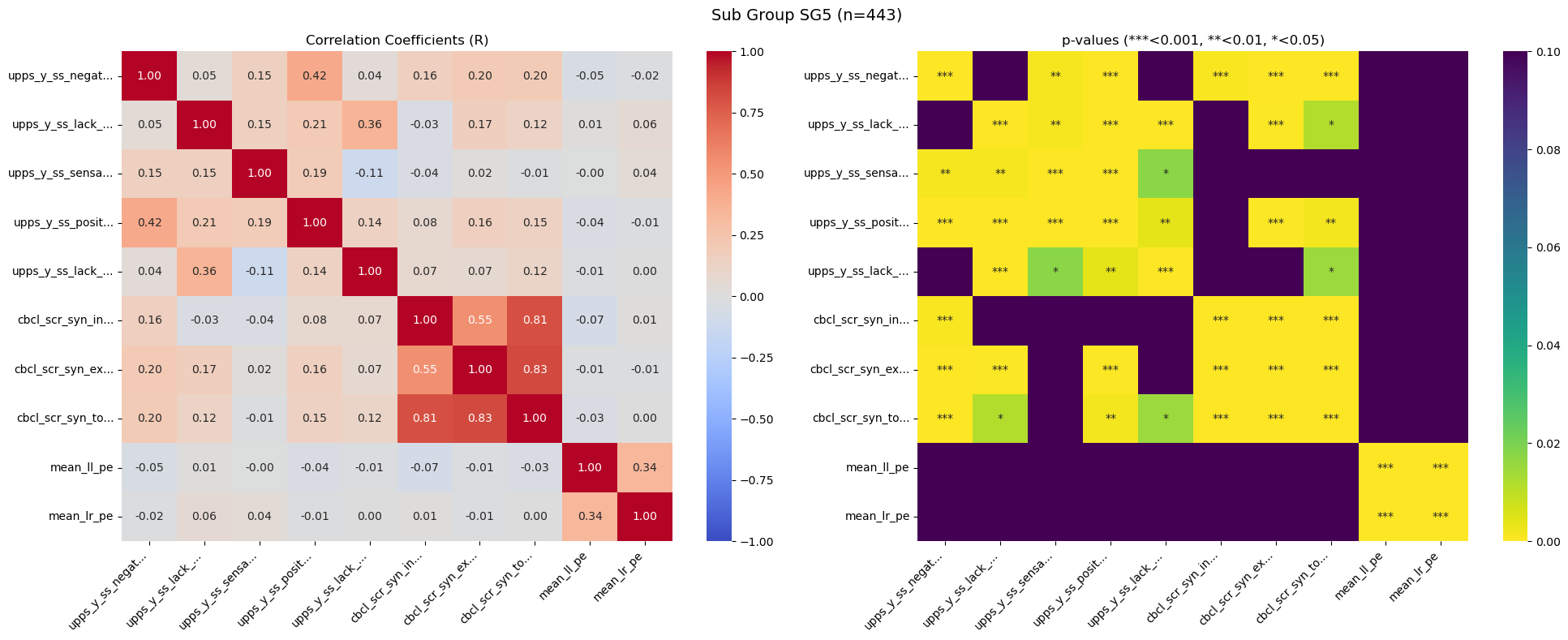


Figure S2E: Correlation between CBCL, UPPS and RPE variables for Subgroup 5

**FHSU – Negative**

Total number of family members with FHSU density - 1052


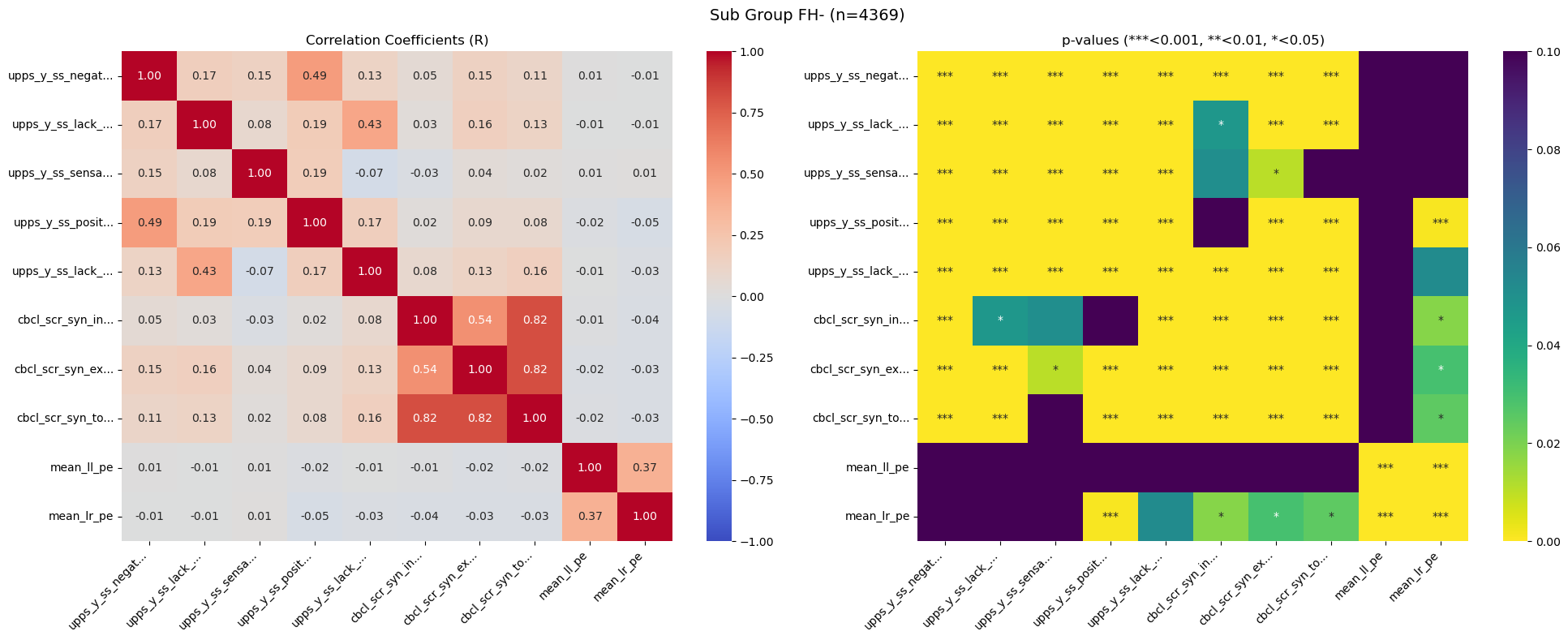


Figure S2F: Correlation between CBCL, UPPS and RPE variables for FHSU-N group.
